# Hints of the Early Jehol Biota: Important Dinosaur Footprint Assemblages from the Jurassic-Cretaceous Boundary Tuchengzi Formation in Beijing, China

**DOI:** 10.1371/journal.pone.0122715

**Published:** 2015-04-22

**Authors:** Lida Xing, Jianping Zhang, Martin G. Lockley, Richard T. McCrea, Hendrik Klein, Luis Alcalá, Lisa G. Buckley, Michael E. Burns, Susanna B. Kümmell, Qing He

**Affiliations:** 1 School of the Earth Sciences and Resources, China University of Geosciences, Beijing 100083, China; 2 Dinosaur Trackers Research Group, University of Colorado Denver, PO Box 173364, Denver, CO 80217, United States of America; 3 Peace Region Palaeontology Research Centre, Box 1540, Tumbler Ridge, British Columbia V0C 2W0, Canada; 4 Saurierwelt Paläontologisches Museum, Alte Richt 7, D-92318 Neumarkt, Germany; 5 Fundación Conjunto Paleontológico de Teruel–Dinópolis, Teruel, Aragón E-44002, Spain; 6 Department of Biological Sciences, University of Alberta, Edmonton, Alberta T6G 2E9, Canada; 7 Institute of Evolutionary Biology, University Witten/Herdecke, Stockumerstr. 10–12, 58454 Witten, Germany; University of Pennsylvania, UNITED STATES

## Abstract

New reports of dinosaur tracksites in the Tuchengzi Formation in the newly established Yanqing Global Geopark, Beijing, China, support previous inferences that the track assemblages from this formation are saurischian-dominated. More specifically, the assemblages appear theropod-dominated, with the majority of well-preserved tracks conforming to the *Grallator* type (sensus lato), thus representing relatively small trackmakers. Such ichnofaunas supplement the skeletal record from this unit that lacks theropods thus far, proving a larger diversity of dinosaur faunas in that region. Sauropods are represented by medium to large sized and narrow and wide-gauge groups, respectively. The latter correspond with earlier discoveries of titanosauriform skeletons in the same unit. Previous records of ornithischian tracks cannot be positively confirmed. Purported occurrences are re-evaluated here, the trackways and imprints, except of a single possible specimen, re-assigned to theropods. Palecologically the Tuchengzi ichnofauna is characteristic of semi-arid fluvio-lacustrine inland basins with Upper Jurassic-Lower Cretaceous deposits in northern China that all show assemblages with abundant theropod and sauropod tracks and minor components of ornithopod, pterosaur and bird tracks.

## Introduction

The Middle-Upper Jurassic Yanliao Biota and the Lower Cretaceous Jehol Biota in northeastern China have been the subject of intense palaeontology research for over a decade due to exceptionally well-preserved fossils such as feathered dinosaurs, angiosperms, primitive mammals, etc. [1, 2].

There is a large temporal gap between the record of the Yanliao Biota and the Jehol Biota [3]. This transition period, however, is nearly equivalent to the age of the Tuchengzi (also called Houcheng) Formation [4]. Thus, the dinosaur fauna of the Tuchengzi Formation has played an important role in discerning the evolution of regional biotas.

No theropod body fossils have been discovered from the Tuchengzi Formation; the only dinosaur body fossils from this unit include the basal ceratopsians *Chaoyangsaurus youngi* and *Xuanhuaceratops niei* [5, 6] and a brachiosaurid sauropod [7]. Therefore, the rich dinosaur tracks from the Tuchengzi Formation represent a significant additional to the faunal information. Dinosaur tracks, primarily those of theropods, are abundant in this unit; the most common are of the grallatorid morphotype [8–18]. Other less common Tuchengzi Formation tracks include *Therangospodus* isp. and *Megalosauripus* isp., didactyl *Menglongipus* tracks, attributed to Dromaeopodidae [14], the bird track *Pullornipes* [19] and possible ornithopod tracks [18].

In July 2011, one of the authors (JZ) visited the Yanqing National Geopark to prepare a Global Geopark application (accepted as a Global Geopark in fall 2013). During the field expedition, abundant dinosaur tracks were discovered. The preliminary research indicated that these tracks may comprise thyreophoran, theropod, ornithopod and sauropod tracks [20]. The sauropod tracks were the first reported from the Tuchengzi Formation. Meanwhile, the Qianjiadian ichno-assemblage is also evidence of flourishing dinosaurian faunas in the Beijing area. Previous evidence of dinosaurs consisted of a fragmentary sauropod rib from the Early Cretaceous Lushangfen Formation of Longtou reservoir, Fangshan District [21]. In August to September, 2013 and April 2014, several of the authors of this paper investigated the Qianjiadian tracksite. Here we present a detailed description of these tracks.

## Distribution of Track Sites

**Fig 1:**
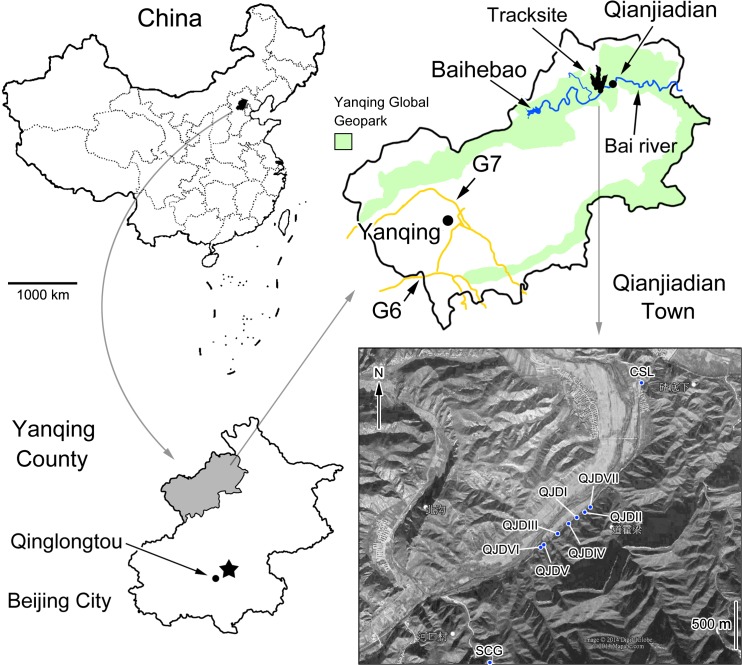
Map and satellite image showing the location of dinosaur tracksites in the Yanqing Global Geopark, Yanqing County, Beijing City, China. QJD = Qianjiadian tracksites I–VI; SCG = Shicaogou tracksite; CSL = Changshouling tracksite.

The Qianjiadian tracksites are situated in the geographical center of the Qianjiadian zone, within the Yanqing Global Geopark in the northern part of Yanqing (Fig 1). The Qianjiadian zone is located in the east of the Geopark, along the Baihe River valley, comprising an area of 108.63 km^2^. The following track-bearing locations are known from this area:

Qianjiadian Tracksite I (QJDI, GPS: 40°40'17.07"N, 116°18'47.66"E) which is divided into upper and lower layers. Tracks described by Zhang et al. (2012) are mostly from the lower layer (QJDILL). The tracks from the upper layer (QJDIUL) had not been documented until 2013. At this time they were made accessible for study by several of the present authors by the use of ropes and rock climbing equipment.

Qianjiadian Tracksite II (QJDII, GPS: 40°40'18.89"N, 116°18'51.12"E) east of Tracksite I, approximately 100 m away. Tracksite II mostly contains seriously-weathered sauropod tracks. No distinct trackway was observed.

Qianjiadian Tracksite III (QJDIII, GPS: 40°40'11.62"N, 116°18'39.18"E) west of Tracksite I, approximately 260 m away. Tracksite III mostly contains undertracks that include about 70 sauropod undertracks and one single theropod undertrack. No distinct trackway was observed.

Qianjiadian Tracksite IV (QJDIV, GPS: 40°40'15.15"N, 116°18'43.90"E) west of Tracksite I, approximately 106 m away. Tracksite IV contains dozens of sauropod undertracks.

Qianjiadian Tracksite V and VI (QJDV, GPS: 40°40'8.89"N, 116°18'35.10"E; QJDVI, GPS: 40°40'8.05"N, 116°18'33.02"E) west of Tracksite I, approximately 442 m away. Tracksites V and VI contain dozens of sauropod undertracks.

Qianjiadian Tracksite VII (QJDVII, GPS: 40°40'20.67"N, 116°18'53.62"E) east of Tracksite I, approximately 180 m away. Tracksite VII contains four sauropod undertracks.

The Shicaogou tracksite (SCG, GPS: 40°39'25.62"N, 116°18'13.08"E) southwest of Tracksite I. The linear distance is approximately 2 km. The tracksite contains a few sauropod and theropod tracks, abundant ripple marks and invertebrate trails.

The Changshouling tracksite (CSL, GPS: 40°41'2.30"N, 116°19'16.53"E) northeast of Tracksite I. The linear distance is approximately 1.5 km. The tracksite contains a few sauropod and theropod tracks.

## Geological Setting

### 1 The Tuchengzi Formation of Yanqing

A series of rift basins developed in the late Mesozoic in northeastern China where volcanism was frequent, such as the Qianjiadian Basin [22]. As an important basin at that time, the deposits of the Tuchengzi Formation mostly accumulated in the Yinshan-Yanshan area around the present northern border of North China, where they then comprised a sequence of typical terrestrial strata formed in an arid and hot environment [22].

**Fig 2:**
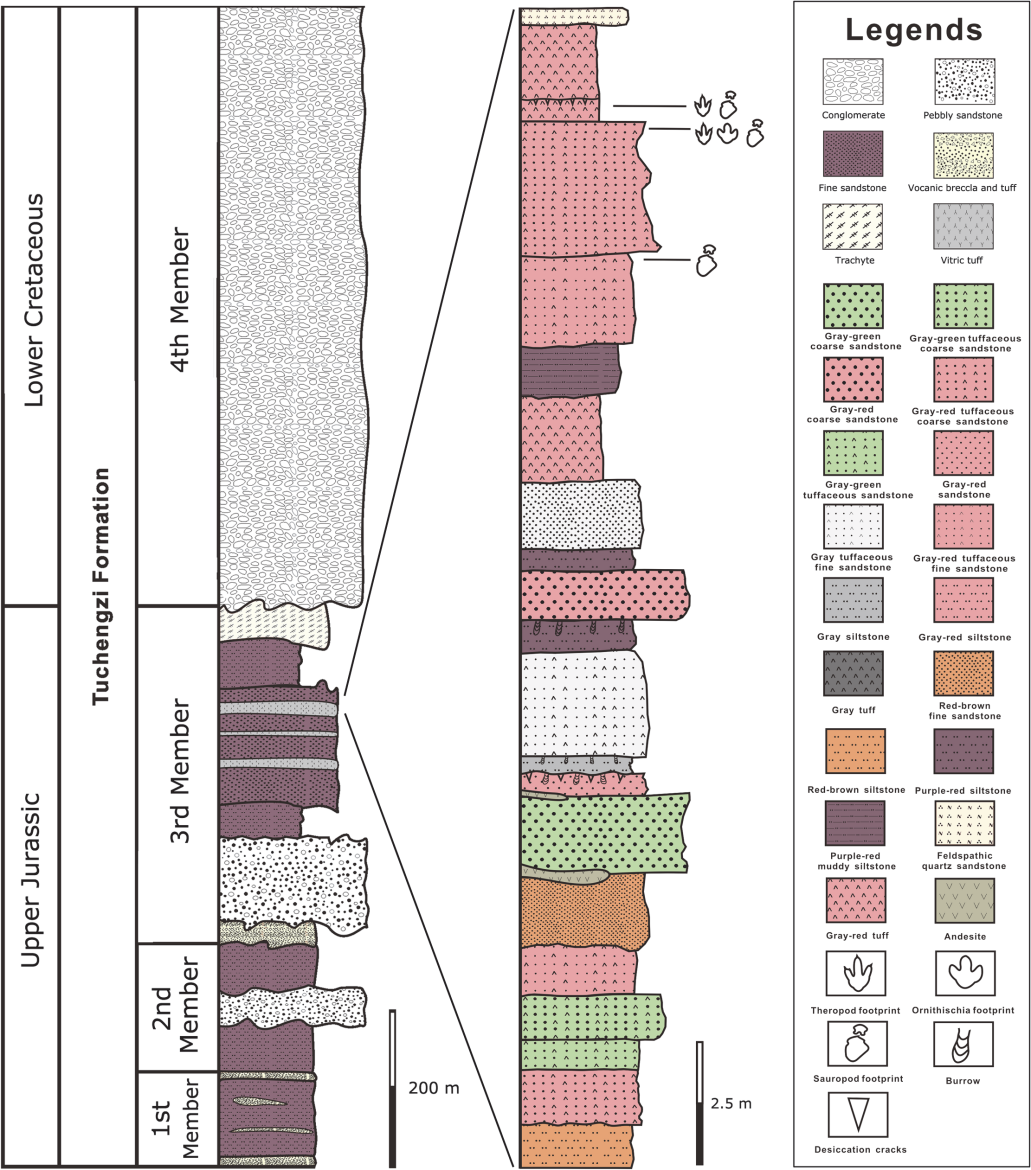
Stratigraphic section of the Tuchengzi Formation and Jurassic-Cretaceous boundary strata as logged at the Qianjiadian tracksites with the position of the track-bearing levels.

In the northern area of Beijing, the Tuchengzi Formation is distributed in the middle-north of Yanqing and exhibits an angular unconformity contact with the Tiaojishan Formation (Upper Jurassic). The Tuchengzi Formation of the Qianjiadian Basin area is completely exposed and can be divided into four members: the First and Second Members belong to the Upper Jurassic, the Third Member located at the boundary of the Upper Jurassic-Lower Cretaceous, and the Fourth Member belonging to the Lower Cretaceous [23]) (Fig 2).

The Qianjiadian tracksite is situated in the upper part of Third Member of the Tuchengzi Formation [20]. The Third Member of the Tuchengzi Formation is a set of purple, greyish-green fluvial facies deposits, dominated by polymictic conglomerate and tuffaceous sandstone, overlain by purple, grayish-green sandstone, siltstone and mudstone. During the deposits of the Tuchengzi Formation, different intensities of volcanic activity affected the basin and created multilayer basic and acidic volcanic rock intercalations of the strata [24]. The major rock types containing tracks are sandstone and sedimentary tuff, among which the lithology of the lower layer of Tracksite I is middle-fine feldspathic litharenite sandstone and the upper layer is tuffaceous sediment. The lithology of Tracksite II is fine, feldspathic, litharenite sandstone containing silt. The analysis of the granularity of the sedimentary rocks indicates that the sedimentary environment represented by the local strata preserving tracks was a lakeshore setting with volcanic debris [24]. From Tracksite II to the upper layer of Tracksite I, the contents of tuff in rocks increase from 5% to 85%, which indicates gradually stronger volcanism [24].

### 2 Age of the Tuchengzi Formation

According to isotope dating analysis, there are two ages proposed for the Tuchengzi Formation: 1, Late Jurassic (e.g. [25], [26]); 2, Late Jurassic–Early Cretaceous (e.g. [22], [27], [28]). The updated Zircon SHRIMP U-Pb dating indicates that the age of the Tuchengzi Formation is 154–137 Ma, from the Late Jurassic Kimmeridgian–Tithonian to the Early Cretaceous Valanginian [4]. The Third Member of the Tuchengzi Formation is situated at the boundary of the Late Jurassic–Early Cretaceous; however, no further dating data have been provided. The age of the Tuchengzi Formation is herein tentatively considered to be close to the Jurassic–Cretaceous boundary.

### 3 Paleoenvironment

The sedimentary environment of the Tuchengzi Formation is fluvial-lacustrine [23]. Characteristic structures include horizontal bedding, mud cracks and incomplete cross bedding. Based on ripple marks, mud cracks and particle size analysis, He [24] considered the Qianjiadian tracksites to represent a shallow-water nearshore deposition. The sedimentary characters of the Upper Jurassic-Lower Cretaceous Tuchengzi Formation in Northern Hebei–Western Liaoning indicate semiarid-arid tropical climate conditions typical of southern Asia [29].

Body fossils document insects, bivalves, conchostracans, fish, and gymnosperms, Abundant invertebrate traces were preserved on the purple sandstones of the Third Member of the Tuchengzi Formation, which include cf. *Monocraterion* isp. and *Scoyenia* isp. [24], with the diameter of the former measuring up to 5 mm. *Scoyenia* isp. preserves irregular rope-like ornamentations on the surface, ranging approximately 2–10 mm in diameter, and the interior burrow showing a backfilling structure. The fillings are muddy deposits from the layer overlying the bioturbated substrate. *Scoyenia* isp. is a typical representative of continental facies [30], and is often associated with vertebrate tracks, as observed at several tracksites in China, such as Jimo, Shandong Province [31].

The Second Member of the Tuchengzi Formation at Xiadelongwan Village, Qianjiadian Town, Yanqing County yields much silicified wood, including *Xenoxylon latiporosum*, *Neocalamites* sp., *Zamites* sp. [32], *Scotoxylon yanqingense* [33]; *Protopiceoxylon extinctum*, *Cupressinoxylon fujeni* [34], [35], [36]. The track area in the Second Member—contains the largest silicified wood flora in North China, all preserved *in situ*. This suggests that the region was heavily forested during the Late Jurassic. However, no fossil plant material has yet been found in the Third Member of the Tuchengzi Formation.

## Materials and Methods

No permits were required to conduct this study. In 2011, researchers used ladders and mechanical devices (“cherry pickers”) to access the tracksite. In 2013, due to the steepness of the bedding planes (dips range 46°–50°, as the top layer of the tracksite was more steep than the lower layer) at tracksites IU, II and III, it was necessary to access the track-bearing surfaces using static climbing ropes and safety harnesses. In order to make accurate maps, tracks were photographed, outlined in chalk, and traced on large sheets of transparent acetate plastic. Videotaping was employed to convert the full size (377 m^2^) tracing maps to a digital format. In addition, a representative area of well-preserved tracks was mapped manually using a chalk grid. Several latex molds (including one of the complete lower surface of Qianjiadian Tracksite I) of representative tracks were made. Detailed tracings of selected individual tracks were made on transparent acetate film, reposited at the University of Colorado (CU). Latex molds, plaster replicas, and most tracings are reposited in the Yanqing Global Geopark, Beijing, China and in the University of Colorado Museum of Natural History collections, Boulder, USA. 3D images of selected tracks were obtained by processing photographs taken with a Canon EOS 70D in Agisoft Photoscan Professional (v 1.0.4). Figures with vertical scale in colour were produced by Cloud Compare (version 2.5.3).

**Fig 3:**
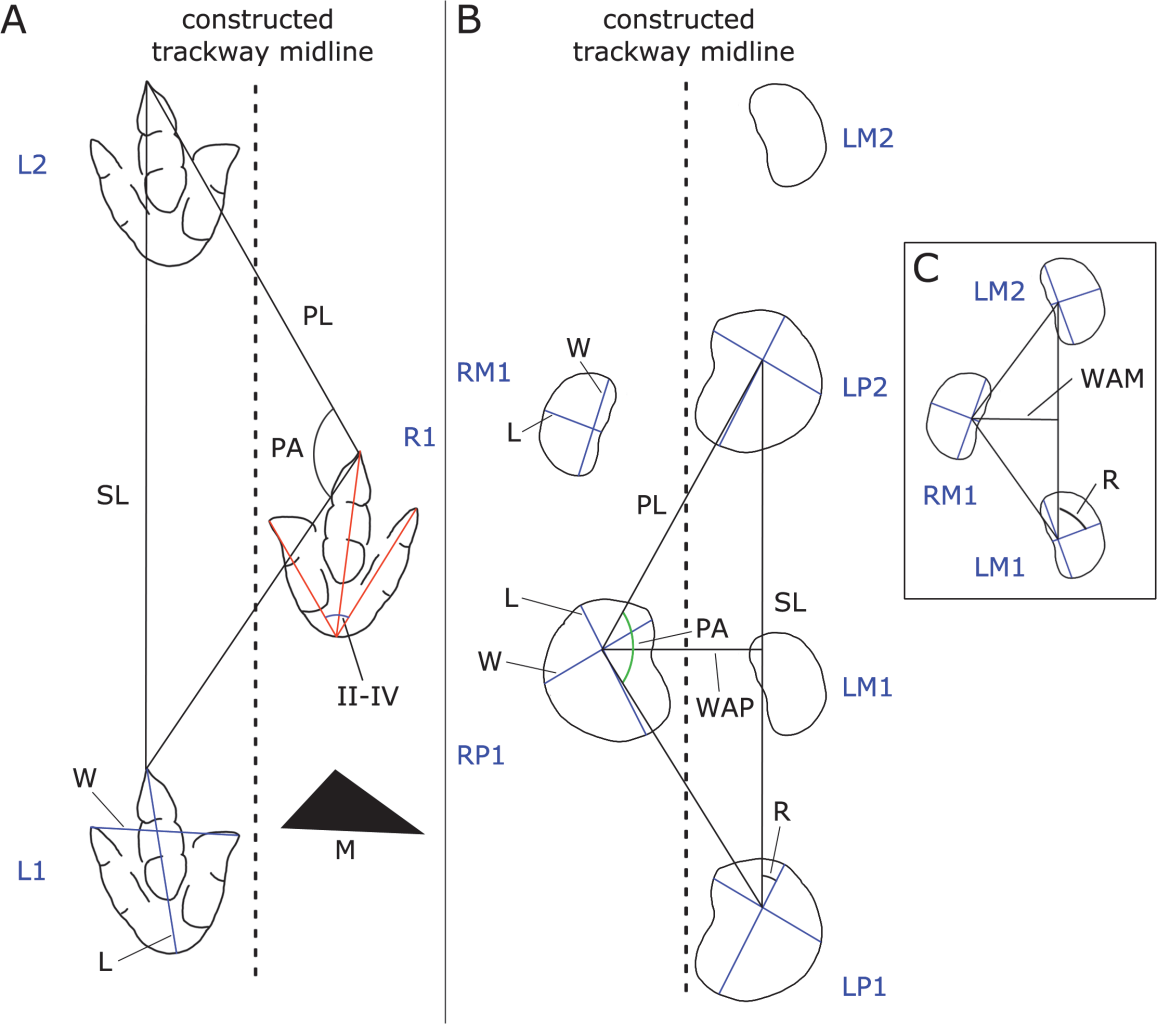
Methods of footprint and trackway measurements used in this study. A, theropod trackway; B and C, sauropod trackways.

For the trackways of sauropods, gauge (trackway width) was quantified for pes and manus tracks using the ratio between the width of the angulation pattern of the pes (WAP) or manus (WAM) and the pes length (P’TL) or manus width (M’TW), respectively [37], [38]. The (WAP/P’TL)-ratio and (WAM/M’TW)-ratio were calculated from pace and stride length, assuming that the width of the angulation pattern intersects the stride at less than a right angle and at the approximate midpoint of the stride [37]. If the (WAP/P’TL)-ratio equals 1.0, the pes tracks are likely to touch the trackway midline. If the ratio is smaller than 1.0, tracks intersect the trackway midline, and are considered to be narrow-gauge (see [39]). A value of 1.0 separates narrow-gauge from medium-gauge trackways, whereas the value 1.2 is arbitrarily fixed between medium-gauge and wide-gauge trackways, and trackways with a value higher than 2.0 are considered to be very wide-gauge [37]. All measuring methods are explained in Fig 3.

For speed calculations from theropod trackways we used the formula of Alexander [40]: *v* = 0.25g^0.5^. SL^1.67^. h^-1.17^, where g = gravitational acceleration in m/sec, SL = stride length, and h = hip height—estimated as 4.5 and 4.9 times foot length, using the ratio for small and large theropods proposed by Thulborn [41]. While McCrea et al. [42] were able to determine a specific calculation based on a known track-maker, Thulborn’s (1990) equation [41] is the most applicable in this case as there is a lack of body fossils of theropods that could be potential track-makers for the Qianjiadian tracks in China [20]. The relative stride length (SL/h) may be used to determine whether the animal is walking (SL/h< = 2.0), trotting (2<SL/h<2.9), or running (SL/h> = 2.9) [40], [41]).

## Qijiadian Tracksites, Yanqing County

### 1. Theropod tracks

#### 1.1. Descriptions and ichnotaxonomic inferences

**Fig 4:**
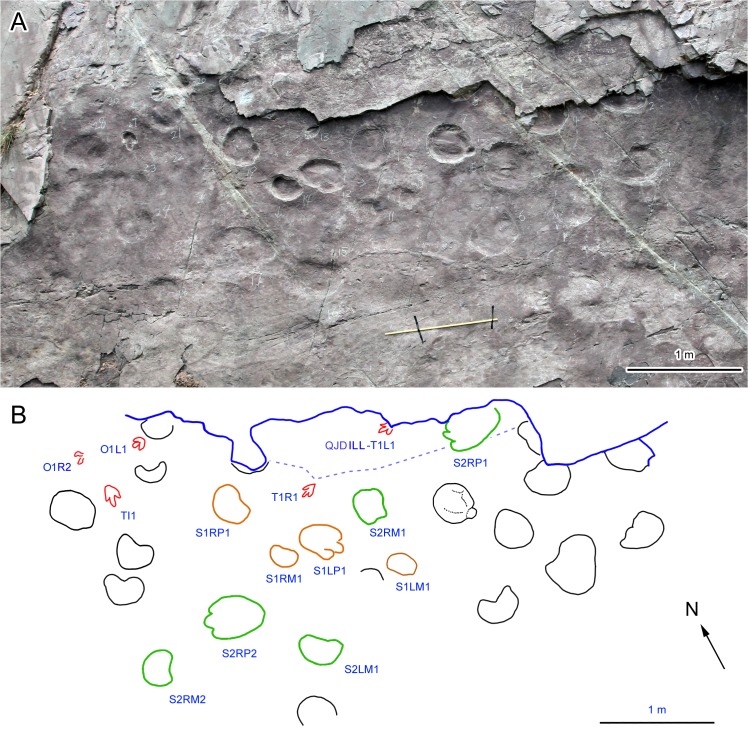
Photograph (A) and interpretative outline drawing (B) of scattered sauropod, theropod and ornithopod tracks at Qianjiadian tracksite IL.

**Fig 5:**
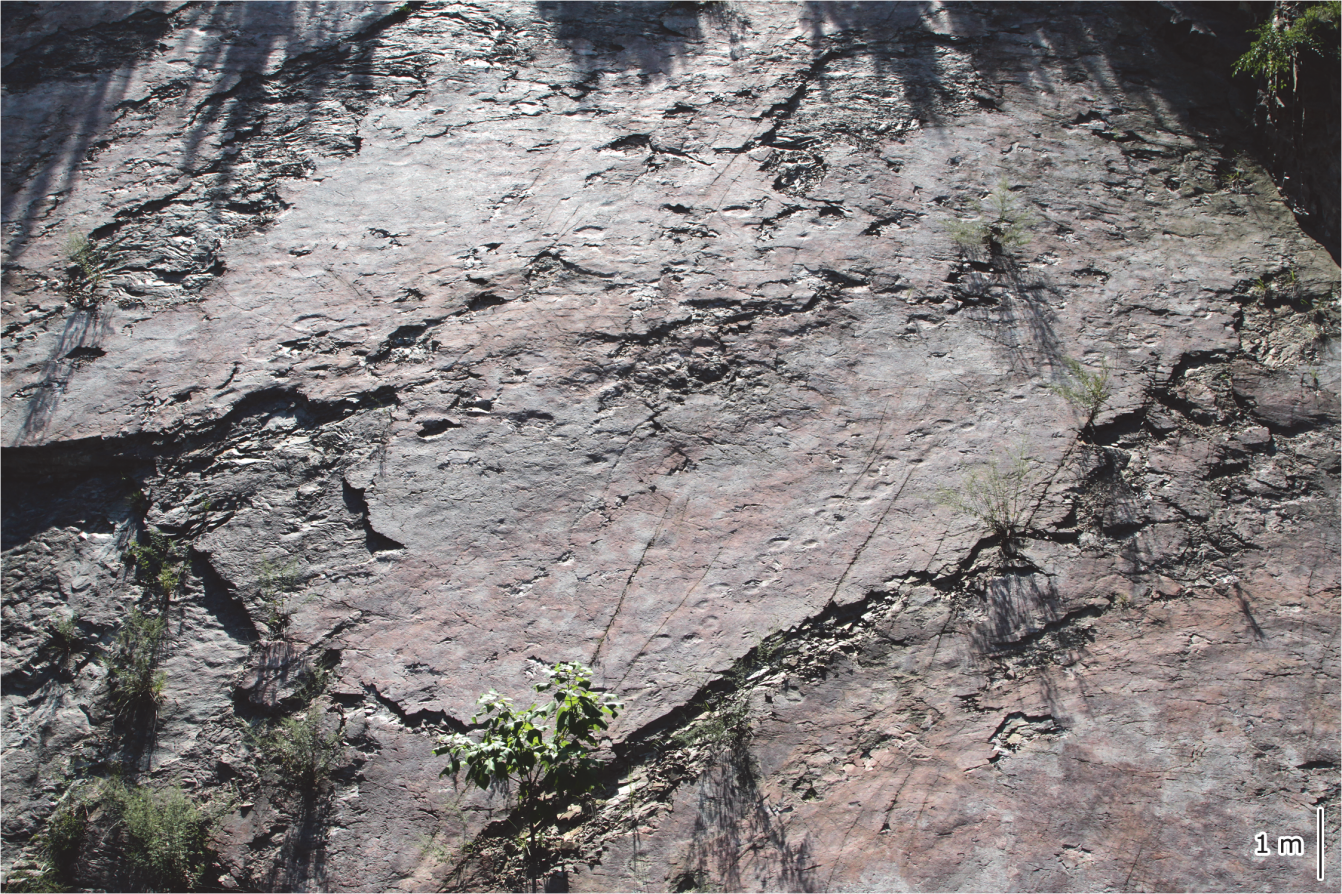
Photograph of Qianjiadian tracksite IU.

**Fig 6:**
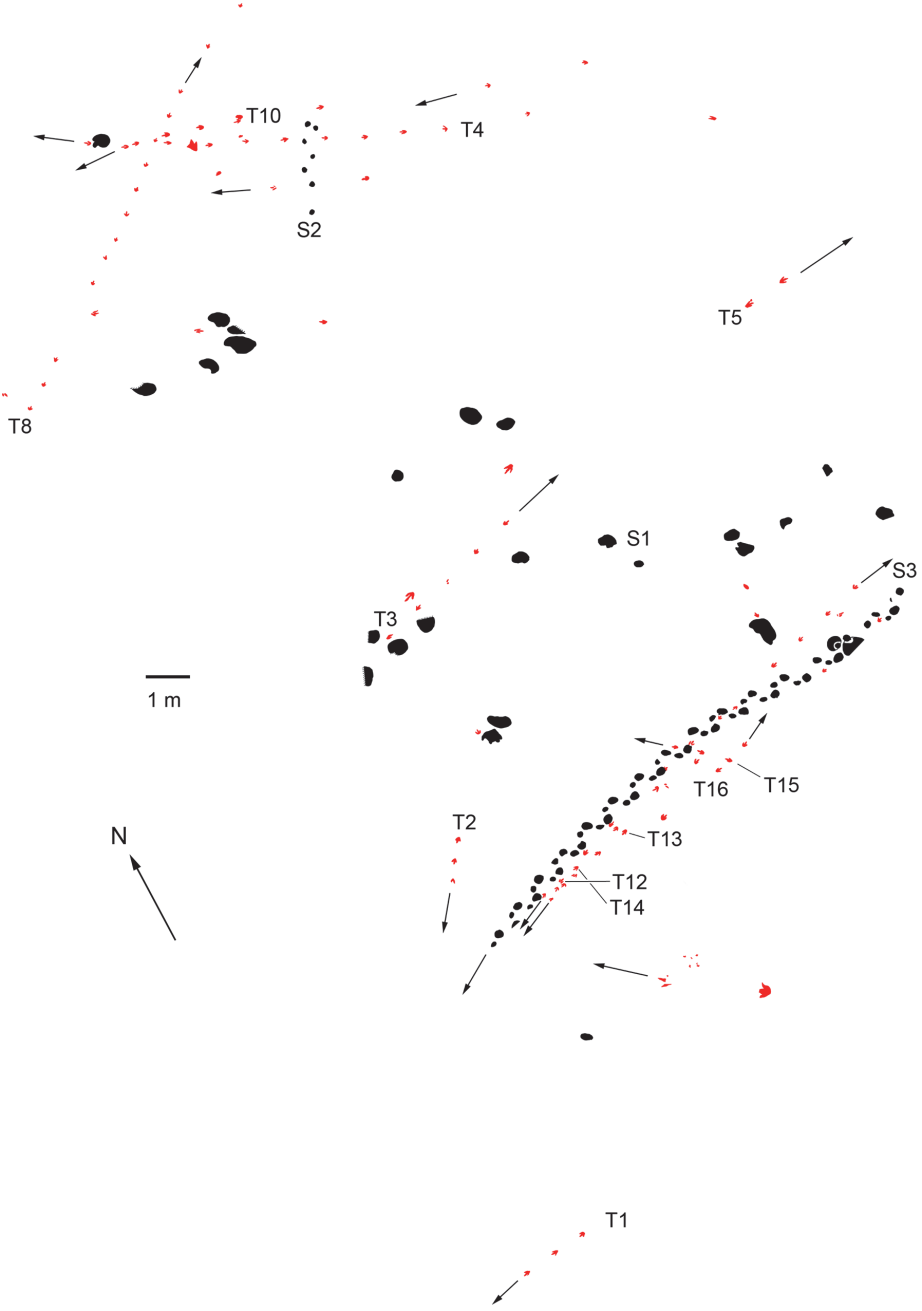
Interpretative outline drawing of track-bearing level at Qianjiadian tracksite IU with trackways and isolated tracks of large sauropods, medium-sized sauropods and theropods.

At the QJDILL site (Fig 4), a trackway consisting of two consecutive tracks (catalogued as T1) and one isolated track (TI1) were discovered, including a moderately well-preserved imprint represented by mold and replica UCM 214.278. QJDIUL (Figs 5 and 6) reveals ~100 tracks, including 12 trackways (catalogued as T1–5, 8, 10, 12–16, total 60 tracks), and 40 isolated tracks. All original specimens remain in the Geopark.

According to Olsen [43], Weems [44], and Lockley [45], theropod tracks can be differentiated on the basis of mesaxony: i.e., the degree to which the central digit (III) protrudes anteriorly beyond the medial (II) and lateral (IV) digits. Carrano and Wilson [46] support the identification of track-makers based primarily on skeletal structures preserved within ichnites.

Carrano and Wilson [46] provide lists of synapomorphies of different dinosaur clades that could possibly be reflected in their fossilized footprints. Some of the Yanqing tracks were produced by functionally tridactyl track-makers possessing claws, characteristics consistent with the assignment to Theropoda. However, Farlow et al. [47] and McCrea et al. [42] have recently commented on the utility and the drawbacks of this method of track-maker identification. As the majority of currently known synapomorphies were present in anatomical areas other than the feet, their use in the identification of track-makers is greatly limited. Fossil footprints were produced by feet that possessed muscles, tendons and integument, which likely masked some osteological features. Footprints are reflections of soft tissue anatomy not generally preserved as body fossils, and thus are potentially useful for establishing new synapomorphies.

The theropod tracks at Yanqing tracksite can be divided into two morphologies: Morphotype A and B; the former is represented by three different track sizes, the latter by two different track sizes.

**Fig 7:**
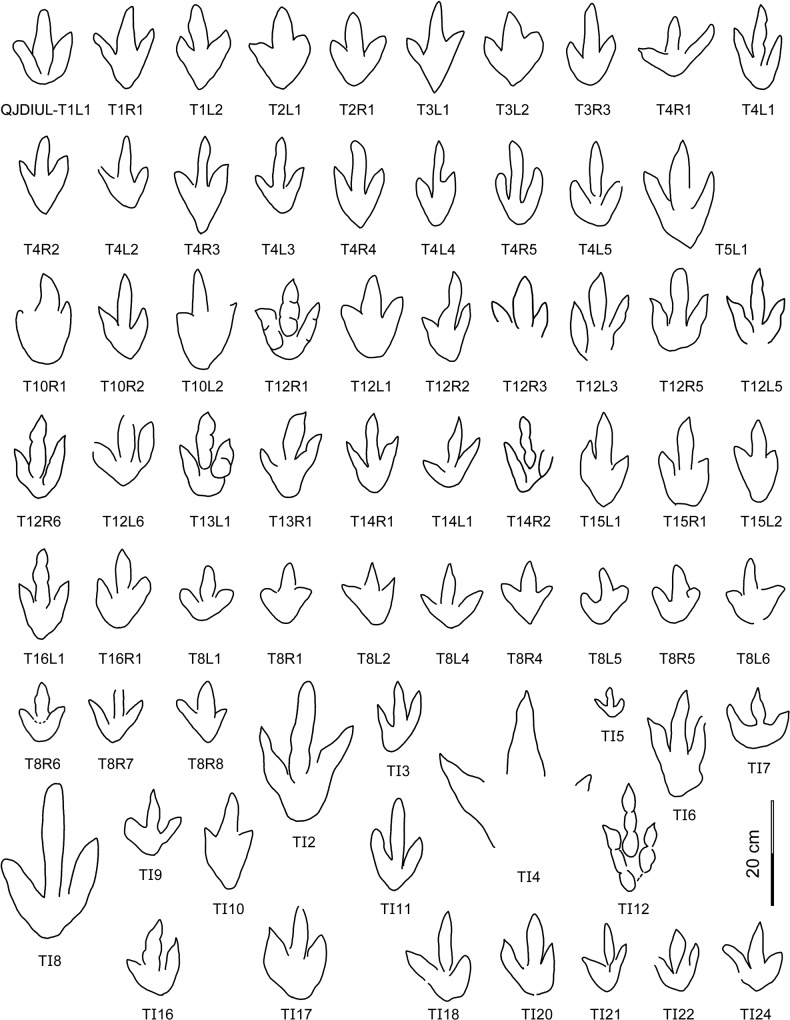
Interpretative outline drawings of theropod tracks at Qianjiadian tracksite IU.

**Fig 8:**
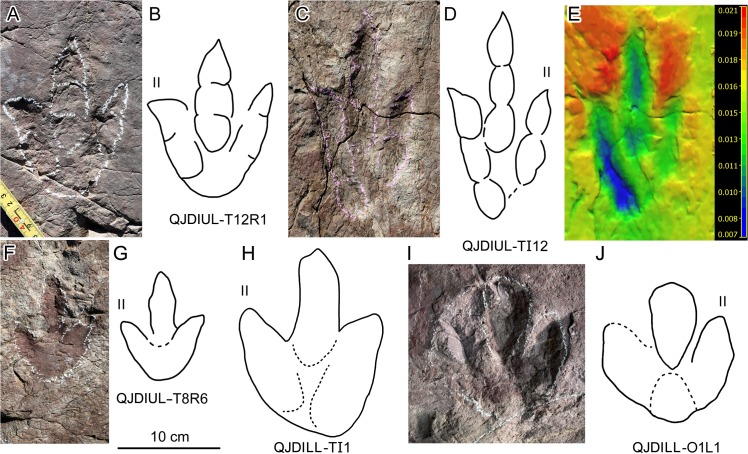
Photograph and interpretative outline drawings of theropod tracks (A–D, F–H) and possible ornithopod track (I–J) at Qianjiadian tracksites IL and IU. (E). 3D model of theropod track QJDIUL-TI12 in C–D.

Morphotype A (Figs 4, 5, 6, 7 and 8, Table 1)—Morphotype A consists of thirty-five natural molds cataloged as QJDIUL-T1, T2, T5, T8, T12, T13, and T14 trackways, which preserved 3, 2, 2, 11, 10, 4, and 3 tracks, respectively, and ten isolated natural molds TI1–5, 7, 9, 16, 17 and 24.

**Table 1:** Measurements (in cm) of tridactyl tracks from the Qianjiadian tracksites, Yanqing, Beijing, China.

Number	TL	TW	II-IV	PL	SL	PA	M	L/W
QJDILL-O1L1	13.8	11.0	61°	59.1	—	—	0.31	1.3
QJDILL-O1R2	—	12.5	—	—	—	—	—	
QJDILL-T1L1	>13.5	9.5	—	88.0	—	—	0.55	—
QJDILL-T1R1	15.5	8.0	44°	—	—	—	0.65	1.9
QJDIUL-T1L1	14.5	11.0	63°	74.0	152.5	175°	0.48	1.3
QJDIUL-T1R1	16.0	11.0	56°	78.0	—	—	0.46	1.5
QJDIUL-T1L2	16.5	9.5	45°	—	—	—	0.70	1.7
Mean	15.7	10.5	55°	76.0	152.5	—	0.55	1.5
QJDIUL-T2L1	15.5	11.0	59°	51.0	98.5	177°	0.58	1.4
QJDIUL-T2R1	14.5	10.0	59°	47.5	—	—	0.52	1.5
QJDIUL-T2L2	—	—	—	—	—	—	—	—
Mean	15.0	10.5	59°	49.3	98.5	177°	0.55	1.5
QJDIUL-T3R1	—	—		—	—	—	—	—
QJDIUL-T3L1	17.0	10.5	52°	88.5	185.5	174°	0.60	1.6
QJDIUL-T3R2	—	—		97.0	—	—	—	—
QJDIUL-T3L2	14.0	10.0	54°	—	—	—	0.45	1.4
QJDIUL-T3R3	15.0	8.0	50°	—	—	—	0.85	1.9
Mean	15.3	9.5	52°	92.8	185.5	174°	0.63	1.6
QJDIUL-T4R1	11.5	13.5	89°	99.5	—	178°	0.28	0.9
QJDIUL-T4L1	16.0	9.0	43°	—	—	—	0.63	1.8
QJDIUL-T4R2	15.0	9.5	46°	92.5	187.0	179°	0.51	1.6
QJDIUL-T4L2	14.0	9.0	59°	94.5	180.0	179°	0.67	1.6
QJDIUL-T4R3	18.0	10	45°	85.0	—	—	0.59	1.8
QJDIUL-T4L3	14.5	9.5	59°	—	—	—	0.73	1.5
QJDIUL-T4R4	16.0	9.0	43°	94.0	191.5	180°	0.65	1.8
QJDIUL-T4L4	16.0	7.0	39°	98.5	—	—	0.97	2.3
QJDIUL-T4R5	16.0	7.5	39°	—	—	—	0.78	2.1
QJDIUL-T4L5	15.5	8.5	55°	—	—	—	0.86	1.8
Mean	15.3	9.3	52°	94.0	186.2	179°	0.67	1.7
QJDIUL-T5L1	20.5	13.0	46°	92.5	—	—	0.48	1.6
QJDIUL-T5R1	—	—	—	—	—	—	—	—
QJDIUL-T8L1	10.5	10.0	81°	64.0	128.0	175°	0.48	1.1
QJDIUL-T8R1	12.0	9.0	74°	64.0	—	—	0.57	1.3
QJDIUL-T8L2	12.0	11.0	62°	—	—	—	0.38	1.1
QJDIUL-T8R4	12.0	9.5	66°	65.0	126.0	172°	0.50	1.3
QJDIUL-T8L4	12.0	12.0	80°	61.0	122.0	177°	0.48	1.0
QJDIUL-T8R5	11.0	9.0	66°	61.0	122.0	177°	0.55	1.2
QJDIUL-T8L5	10.0	9.0	70°	62.0	126.0	180°	0.51	1.1
QJDIUL-T8R6	10.5	8.0	68°	64.5	127.5	176°	0.58	1.3
QJDIUL-T8L6	12.0	11.0	82°	63.0	—	—	0.55	1.1
QJDIUL-T8R7	11.0	10.0	65°	—	124.0	—	—	1.1
QJDIUL-T8L7	—	—	—	—	—	—	—	—
QJDIUL-T8R8	11.5	9.5	66°	—	—	—	0.50	1.2
Mean	11.3	9.8	71°	63.1	125.1	176°	0.51	1.2
QJDIUL-T10L1	—	8.5	—	86.0	163.5	176°	—	—
QJDIUL-T10R1	17.0	9.0	46°	78.0	149.0	178°	0.64	1.9
QJDIUL-T10L2	18.5	11.0	47°	70.5	—	—	0.70	1.7
QJDIUL-T10R2	16.5	8.5	51°	—	—	—	0.76	1.9
Mean	17.3	9.3	48°	78.2	156.3	177°	0.70	1.8
QJDIUL-T12R1	14.5	9.5	61°	92.0	187.0	177°	0.40	1.5
QJDIUL-T12L1	15.5	11.0	56°	95.0	—	—	0.44	1.4
QJDIUL-T12R2	16.0	9.5	52°	—	184.0	—	0.73	1.7
QJDIUL-T12L2	—	—	—	—	—	—	—	—
QJDIUL-T12R3	—	9.0	—	91.0	183.0	177°	0.52	—
QJDIUL-T12L3	16.0	10.5	—	92.0	—	—	0.52	1.5
QJDIUL-T12R4	11.0	10.0	—	—	189.0	—	—	1.1
QJDIUL-T12L4	—	—	—	—	—	—	—	—
QJDIUL-T12R5	14.5	11.5	59°	92.0	186.0	179°	0.58	1.3
QJDIUL-T12L5	13.0	10.0	62°	93.5	188.0	180°	0.57	1.3
QJDIUL-T12R6	14.5	10.0	50°	94.0	—	—	0.52	1.5
QJDIUL-T12L6	>13.0	11.0	—	—	—	—	—	—
Mean	14.2	10.2	57°	92.8	186.2	178°	0.54	1.4
QJDIUL-T13L1	16.0	9.5	49°	83.0	160.0	177°	0.64	1.7
QJDIUL-T13R1	16.0	11.5	57°	79.0	160.0	179°	0.43	1.4
QJDIUL-T13L2	>11.0	11.5	—	81.0	—	—	—	—
QJDIUL-T13R2	>10.5	—	—	—	—	—	—	—
Mean	16.0	10.8	53°	81.0	160.0	178°	0.54	1.6
QJDIUL-T14R1	15.0	10.0	51°	52.0	103.0	153°	0.61	1.5
QJDIUL-T14L1	13.0	11.5	78°	53.0	—	—	0.43	1.1
QJDIUL-T14R2	13.0	8.0	—	—	—	—	—	1.6
Mean	13.7	9.8	65°	52.5	103.0	153°	0.52	1.4
QJDIUL-T15L1	18.0	8.0	39°	68.5	131.5	177°	0.79	2.3
QJDIUL-T15R1	17.0	9.0	50°	63.0	—	—	0.80	1.9
QJDIUL-T15L2	15.5	8.0	41°	—	—	—	0.72	1.9
Mean	16.8	8.3	43°	65.8	131.5	177°	0.77	2.0
QJDIUL-T16L1	17.0	9.5	48°	90.0	—	—	0.75	1.8
QJDIUL-T16R1	15.0	9.5	52°	—	—	—	0.60	1.6
Mean	16.0	9.5	50°	90.0	—	—	0.68	1.7
QJDILL-TI1	18.5	13.2	55°	—	—	—	0.48	1.4
QJDIUL-TI2	26.0	17.0	52°	—	—	—	0.53	1.5
QJDIUL-TI3	13.0	8.5	41°	—	—	—	0.37	1.5
QJDIUL-TI4	>30.0	28.0	—	—	—	—	0.51	—
QJDIUL-TI5	5.5	5.0	73°	—	—	—	0.42	1.1
QJDIUL-TI6	20.0	11.0	40°	—	—	—	—	1.8
QJDIUL-TI7	11.5	11.0	75°	—	—	—	0.33	1.0
QJDIUL-TI8	28.5	17.0	57°	—	—	—	0.67	1.7
QJDIUL-TI9	12.5	10.0	78°	—	—	—	0.52	1.3
QJDIUL-TI10	18.0	9.0	44°	—	—	—	0.84	2.0
QJDIUL-TI11	17.5	8.0	43°	—	—	—	0.79	2.2
QJDIUL-TI12	20.0	10.0	42°	—	—	—	0.89	2.0
QJDIUL-TI15	12.0	10.0	—	—	—	—	—	1.2
QJDIUL-TI16	14.0	10.5	60°	—	—	—	0.53	1.3
QJDIUL-TI17	17.5	10.5	42°	—	—	—	0.56	1.7
QJDIUL-TI18	17.5	11.0	62°	—	—	—	0.70	1.6
QJDIUL-TI20	15.0	9.5	47°	—	—	—	0.66	1.6
QJDIUL-TI21	13.0	9.0	57°	—	—	—	0.60	1.4
QJDIUL-TI22	12.0	8.5	55°	—	—	—	—	1.4
QJDIUL-TI23	17.5	—	—	—	—	—	—	—
QJDIUL-TI24	13.0	10.5	68°	—	—	—	0.44	1.2
SCG-TI1	10.8	5.3	27°	—	—	—	—	2.0
CSL-TI1	28.0	20.3	61°	—	—	—	0.46	1.4

Abbreviations: TL: Track length; TW: Track width (measured as the distance between the tips of digits II and IV); II-IV: angle between digits II and IV; PL: Pace length; SL: Stride length; PA: Pace angulation; M: Mesaxony; L/W: Track length/track width.

These tracks are divided into small (length 5.5 cm), medium-sized (length ~11–20 cm) and large (length 26–30 cm) tridactyl tracks, with an average TL/TW ratio of 1.5. Morphotype A is characterized by weak to moderate mesaxony (average 0.51, ranges between 0.33 to 0.56, N = 37), which is typical for footprints of the ichno- or morphofamily Eubrontidae Lull 1904 [48].

Morphotype A resembles the classic theropod footprint genera *Eubrontes*, *Anchisauripus* and *Grallator* from the Late Triassic-Early Jurassic, but it commonly has a wider divarication of digits II–IV (average 58°) compared with *Eubrontes* (10°–40°) [49]. The average pace angulation is 172°.

The best-preserved track of Morphotype A is QJDIUL-T12R1 (Figs 7, 8A and 8B). It is a tridactyl right pes imprint with a TL/TW ratio of 1.5. Digit II is robust and the shortest, digit IV is the longest. Each digit terminates in a sharp claw mark. Digits II, III and IV have 2, 3, and 3 phalangeal pads, respectively. The borders of the digital pads and metatarsophalangeal pad of digit IV are indistinct. The divarication between digits II and III (36°) is larger than that between digits III and IV (25°). The metatarsophalangeal pad is well-developed, located at the proximal end of the long-axis of digit III.

Morphotype A track QJDILL-TI1 (Fig 8H) was incompletely exposed. It was originally attributed to ornithopod track-makers based on the blunt, rounded digits [20]. After further preparation, the track is considered here to be of theropod affinity. The stout digital prints are probably caused by preservation in soft sediments [50].

At the Qianjiadian tracksites, the largest theropod track is QJDIUL-TI4 (Fig 7). It is poorly-preserved and lacks the posterior region. However, the imprint is at least 30 cm in length. The three claw marks are sharp. Another incomplete large track (QJDIUL-TI4.2) is located along the same axis as TI4, with an interval of approximately 2 m. Presumably it is the ipsilateral track constituting one single stride along with TI4. The smallest theropod track is QJDIUL-TI5 (Fig 7) with a length of 5.5 cm, with a wide divarication of digits II–IV (73°), and consistent with T12R1 in morphology. TI5 and another incomplete small track (QJDIUL-TI5.2–5) might constitute one single pace; the interval is 24 cm.

T8 is a trackway of a small-sized individual with a mean pes length of 11.3 cm. Compared with other Morphotype A tracks, the mean TL/TW ratio (1.2) is lower, the mean digits II–IV divarication (71°) is wider, but the trackways are seriously weathered. The mean mesaxony is 0.51, which is similar to Morphotype A. The lower TL/TW ratio and wider divarication are probably characteristics of juvenile trackmakers of Morphotype A.

**Fig 9:**
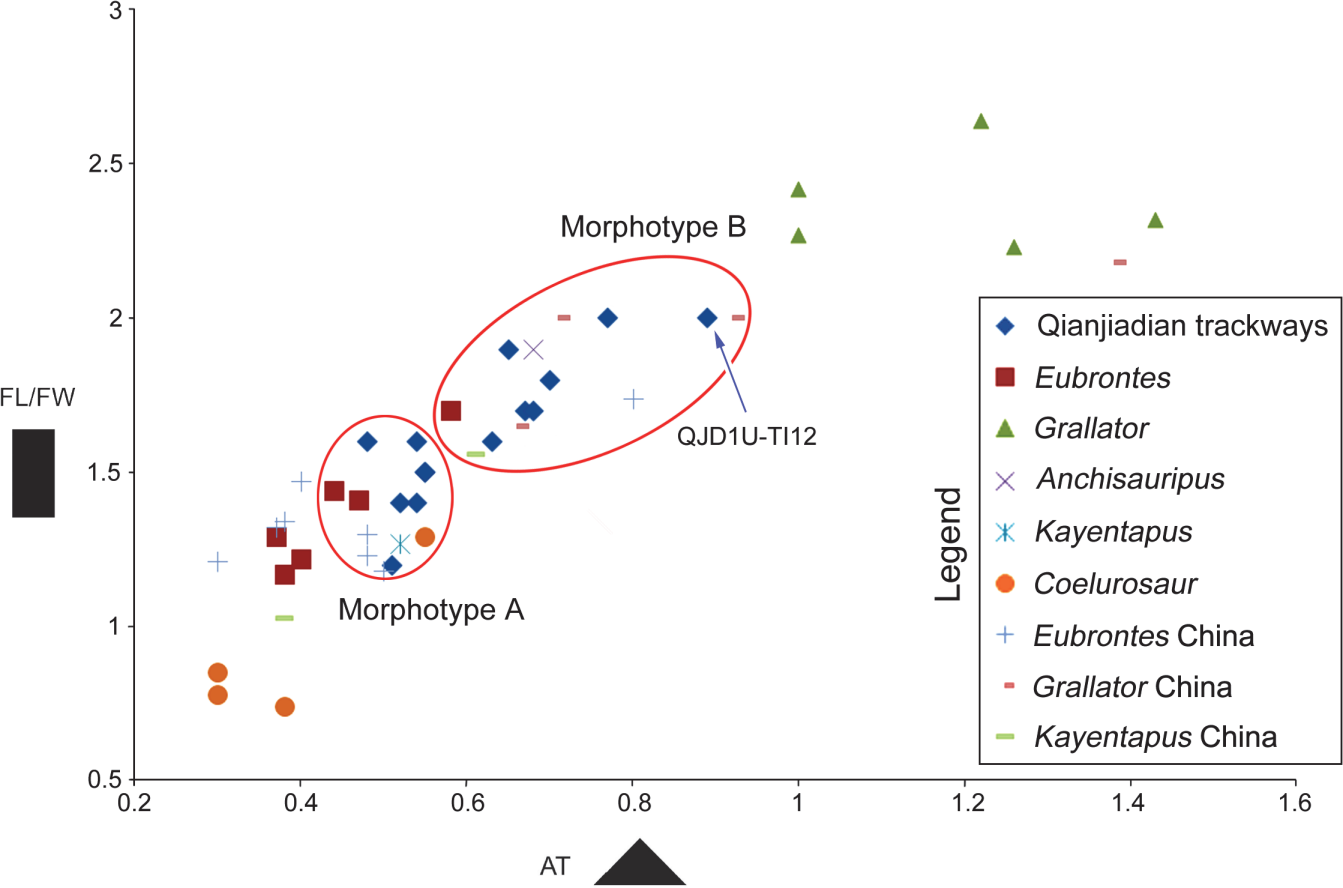
Bivariate analysis of the length/width ratio vs. AT (anterior triangle length-width ratio) of Qianjiadian theropod tracks and other tridactyl theropod ichnotaxa [45].

Based on a bivariate analysis of the TL/TW ratio vs. AT (anterior triangle length-width ratio) of the Qianjiadian theropod tracks and other tridactyl theropod ichnotaxa [45], a natural break in at mesaxony 0.56 (Fig 9) was identified.

Morphotype A is similar to the type specimens of *Eubrontes* [44] and Chinese *Eubrontes* type tracks [45]. Most of the coelurosaur tracks have lower mesaxony (0.30–0.38). However, most Morphotype A tracks (except TI4) are smaller than typical *Eubrontes* type tracks (>25 cm; [49]) in size. In addition, all Morphotype A tracks lack discernable digit II metatarsophalangeal pads. Thus, Morphotype A are herein tentatively attributed to cf. *Eubrontes*.

Morphotype B (Figs 4, 7, 8 and Table 1) consists of thirty-six natural molds cataloged as QJDILL-T1, QJDIUL-T3, T4, T10, T15, and T16 trackways, preserving 2, 4, 10, 4, 3, and 2 tracks respectively, and eleven isolated natural molds: TI6, 8, 10, 11, 12, 15, 18, 20–23.

These tracks are divided into medium-sized (length ~12–20 cm) and large-sized (length 28.5 cm) tridactyl tracks, with an average TL/TW ratio of 1.7. The divarication of digits II–IV of Morphotype B is also smaller (average 49°) than in Morphotype A. The average pace angulation is 177°. Morphotype B is characterized by stronger mesaxony (average 0.69, ranges between 0.59 to 0.97, N = 26) compared with Morphotype A, which is more close to the ichno- or morphofamily Grallatoridae Lull 1904 [48].

Among these tracks the best-preserved is QJDIUL-TI12 (Fig 8C–8E **)** It is a tridactyl left pes, with a TL/TW ratio of 2 and stronger mesaxony (0.89). Digit II is the shortest and, digits III and IV are almost equal in length. Each digit has a sharp claw mark. Digits II and III have 2 and 3 phalangeal pads respectively. The borders of the digital pads of digit IV are indistinct. The divarication between digits II and III (19°) is smaller than that between digits III and IV (23°). The metatarsophalangeal pad of digit IV is developed close to the axis of digit III.

The largest track of Morphotype B is QJDIUL-TI8 (Fig 7), which is 28.5 cm in length and similar to TI12 in morphology, whereas the mesaxony (0.67) is weaker. Other Morphotype B tracks are smaller, not longer than 20 cm. TI8 is presumed as an extramorphological variation of comparably larger tracks of Morphotype A.

Morphotype B tracks are similar to other *Grallator* type tracks reported from China (Lockley, 2009: Fig 8 [45]); however, the mesaxony is weaker than reported for the North American *Grallator* [49]. Although the small sample size makes it difficult to identify systematic features, Morphotype B are herein referred to *Grallator* isp.

#### 1.2 Comparisons and discussion

There have been at least nine tracksites yielding theropod tracks from the Tuchengzi Formation, many of which are similar to the Qianjiadian theropod tracks.

Sijiazi tracksites, Yangshan area, Liaoning Province [8], [9], [10], [51], [12]. Yabe et al. [8] described the footprints as a new ichnogenus and ichnospecies *Jeholosauripus ssatoi*. However, *Jeholosauripus* was considered a junior synonym of *Grallator* by Zhen et al. [51]. There are nearly 4000 tracks with lengths ranging between 7–12 cm [8], [51], and their orientations are relatively consistent. Matsukawa et al. [12] illustrated the track distribution at several sites in the Sijiazi tracksite complex where they mapped more than 1000 tracks, and provided detailed illustrations of four tracks ([12]: Fig 2). The mean TL/TW ratio of the four well-preserved tracks is 1.7 (between 1.6 to 1.8, N = 4), the mean TL/TW ratio of the anterior triangle is 0.71 (ranges between 0.63 to 0.75, N = 4). This stronger mesaxony resembles Qianjiadian Morphotype B, and both are identified here as *Grallator* isp.

Sijiaban tracksite I, Nanbajiazi (Nan Pachazu) *Township*, *near Beipiao City*, Liaoning Province *(Fujita et al*., *2007)*. *Fujita et al*. *(2007) distinguished three different size classes*: Type A has an average length of 4.5 cm (over 100 tracks), Type B has an average length of 13.4 cm (three tracks), and Type C has an average length of 16.7 cm (14 tracks). Walking orientations of the various types are relatively consistent [13]. For B1 and C14 ([13]: Fig 6), which are well-preserved specimens of *Grallator*, the TL/TW ratio ranges between 1.7 and 1.8, and the AT ranges between 0.5 and 0.55. These characteristics fall in between Qianjiadian Morphotype A and B, with a mesaxony similar to Morphotype A and the TL/TW ratio similar to Morphotype B. However, both B1 and C14 have a distinctive, relatively swollen metatarsophalangeal region, positioned in line with the long axis of digit III, which makes B1 and C14 more similar to *Jialingpus* [18] than *Grallator* [18].

Sijiaban tracksite II, Nanbajiazi County, near Beipiao City, Liaoning Province [11]. Zhang et al. [11] divided the tracks into three size classes: 1–10 cm (eight tracks), 10–20 cm (30 tracks), and 20–30 cm (nine tracks). The walking orientations of the tracks are consistent across all three size ranges *but* no detailed descriptions were provided by Zhang et al. [11].

Kangjiatun tracksite near Kangjiatun Village, Beipiao City, Liaoning Province [19]. The Kangjiatun tracksite is dominated by bird tracks. The bird tracks are attributed to *Pullornipes aureus*, *Aquatilavipes* or cf. *Aquatilavipes*. *Grallator* type tracks were not described in detail.

Shibanwo tracksite, Majiagou village, Chengde city, Hebei Province [52], [12]. Young [52] described these footprints as *Jeholosauripus ssatoi*. *J*. *ssatoi* should be attributed to *Grallator ssatoi* [51]. Matsukawa et al. [12] considered the tracks from this site to belong to theropods, hypsilophodontids, and birds (*Aquatilavipes*).

Nanshuangmiao tracksite in Chengde County, northern Hebei Province [15]. Tracks from this site (all in all nine imprints) are poorly-preserved. No discernable trackways have been observed. Among these, eight tracks range between 12.3 cm and 18.5 cm in length. The largest track is 28.8 cm in length. Sullivan et al. [15] assigned the material to *Anchisauripus*, however, it is insufficient for a confident identification. The AT is 1.00 (IVPP VC 15815 G) and 0.63 (IVPP VC 15815 B and F). Presumably it represents two different morphologies of theropod tracks, as inferred for the Qianjiadian tracksite, and possibly two different ichnospecies [18].

Siliang tracksite, Chicheng County, Hebei Province [14], [17]. Most *Grallator* type tracks from the Nijiagou area are poorly-preserved. They have an average length of 20 cm (roughly 40 tracks). In addition the inferred deinonychosaurian tracks *Menglongipus* and two indistinct larger theropod tracks with lengths of 59 cm and 63 cm were also discovered at the Silang site [14]: see Lockley et al. [53] for further discussion of the range of variation in deinonychosaurian tracks.

Luofengpo tracksite, Chicheng County, Hebei Province [16]. Xing et al. [16] described 169 theropod tracks from the Luofengpo tracksite, among which 163 well-preserved tracks were assigned to *Therangospodus* isp., and one large-sized track to *Megalosauripus* isp. The *Therangospodus* assignment was based on the morphology: medium-sized (averaging 28 cm in length), elongate, asymmetric theropod tracks with coalesced, elongate, oval digital pads that are not subdivided into discrete phalangeal pads, and with an AT of 0.55 [54], [18]. However, based on Iberian Range (Spain) *Therangospodus oncalensis* materials, Castanera et al. [55] suggested that the producer of the tracks from Spain was an ornithopod rather than a theropod. However, these authors acknowledge that the type of *Therangospodus* (*T*. *pandemicus*) from the Upper Jurassic of Utah is definitively theropodan. It indicates that the Chinese materials, which were assigned to *Therangospodus*, need to be re-considered in the light of our changing view of variation within the ichnogenus and its stratigraphic range. The Luofengpo tracks can be attributed to theropods based on the sharp claw mark, the pad formula 0-2-3-4-0, and the nearly straight-line trackway. Taking the best-preserved LF 1 as an example, the track has a TL/TW ratio of 1.3 and AT of 0.54. These characteristics are similar to the Qianjiadian Morphotype A and suggest that both probably belong to the same ichnogenus.

Shangyi tracksite, Zhangjiakou City, Hebei Province [18]. Xing et al. [18] assigned fifty-four Shangyi theropod tracks to *Therangospodus* isp., and fifteen tracks with a low TL/TW ratio (1.2–1.3) to cf. Ornithopoda. For the Shangyi theropod tracks, the mean TL/TW ratio is 1.7 (N = 38), and the AT is 0.69 (N = 36), which is close to Qianjiadian Morphotype B. The Shangyi theropod tracks also lack a developed metatarsophalangeal region. This is similar to the condition seen in the Qianjiadian Morphotype B. However, most Shangyi theropod tracks are stouter than Qianjiadian Morphotype B, which probably suggests the morphological difference in the pes of *Grallator* type trackmakers.

In addition, a few *Grallator* isp. were discovered in the Yixian Formation of the Sihetun tracksite [14]. The size of tracks at the Sihetun site (13–15.5 cm) and the high mesaxony are similar to Qianjiadian Morphotype B. Both have a relatively wide divarication (mean 53° and 49° in Morphotype B, respectively), that suggests that the local theropod tracks are consistent in morphology during the Late Jurassic–Early Cretaceous.

Based on TL/TW ratio and mesaxony, there are at least two types of theropod tracks (*Eubrontes* type and *Grallator* type) representing tendencies towards a polarity between the wider and narrow morphotypes. Additionally, there are also some tracks in between the two types. The theropod tracks have a wide divarication (generally larger than 40°), commonly larger than typical *Grallator* tracks in North America (10°–30°). However, currently-discovered *Grallator* tracks from the Early Jurassic from southwest China also have a wide divarication, such as *Grallator microiscus* [56]. Generally, *Grallator* type tracks from China show a wider divarication that could be due to differences of the pes anatomy based on regional differences between China and North America, to different preservation, or provincial ichnotaxonomy pertaining to the actual material studied. There is diversity in the sizes of tracks discovered within the same areas. These tracks may be similar in morphology, but it does not necessarily suggest different ontogenetic ages of the same trackmaker. Considering that the evidence for the radiation of theropods in northeastern China is first recognized near the Middle Jurassic-Upper Jurassic boundary [57, 58](Hu et al., 2009; Xu et al., 2010), and there are diverse theropod associations in the Yixian Formation, consisting of compsognathids, dromaeosaurids, troodontids, oviraptorosaurs, therizinosauroids and tyrannosauroids [59], this diversity is more likely the result of various trackmaking species. Xing et al. [14] proposed that the foot morphology capable of producing *Grallator* tracks may therefore have been widely distributed in small–medium sized theropods (other than dromaeosaurids and troodontids) from the Yixian Formation.

*Eubrontes*, together with *Grallator*, *Kayentapus* and *Anomoepus*, are typical components of the Lower Jurassic ichno-associations from North America [60], [49]. However, in China, they occur not only in Lower Jurassic formations, but also in Middle and even Upper Jurassic and Lower Cretaceous strata (see [12] for comment on the high proportion of theropod tracks in the Cretaceous of China). Thus, theropod tracks from the Jurassic of China presently cannot be used for detailed biostratigraphic purposes [61]. Given the broad stratigraphic interval in which *Eubrontes* and *Grallator* isp. tracks occur in China, the Tuchengzi Formation tracks are not conclusive evidence to determine whether the stratum are Upper Jurassic or Lower Cretaceous.

### 2 Sauropod tracks

#### 2.1 Descriptions and ichnotaxonomic inferences

**Fig 10:**
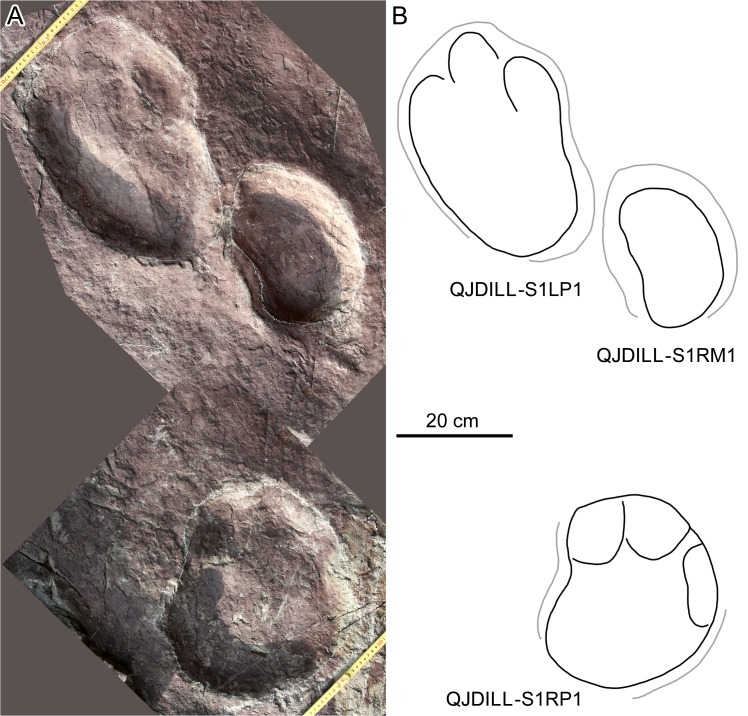
Photograph (A) and interpretative outline drawing (B) of sauropod trackway segment QJDILL.

**Fig 11:**
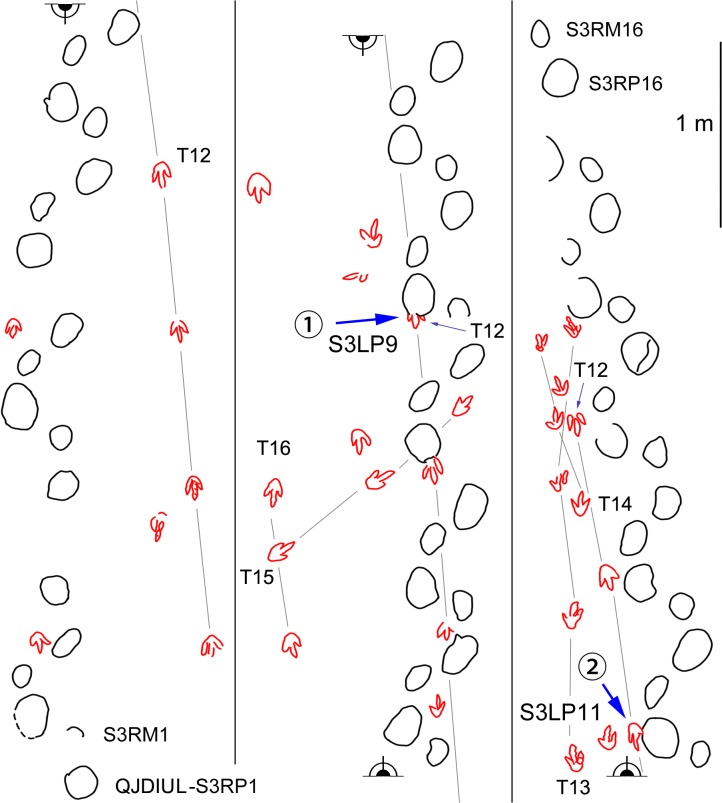
Interpretative outline drawing of sauropod and theropod trackways at Qianjiadian tracksite IU.

**Fig 12:**
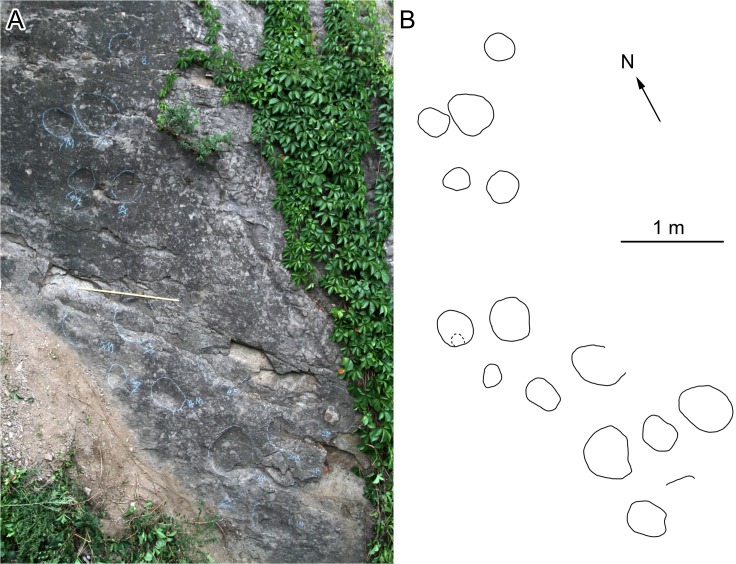
Photograph (A) and interpretative outline drawing (B) of scattered sauropod tracks at Qianjiadian tracksite II. Length of scale in A = 1m.

**Fig 13:**
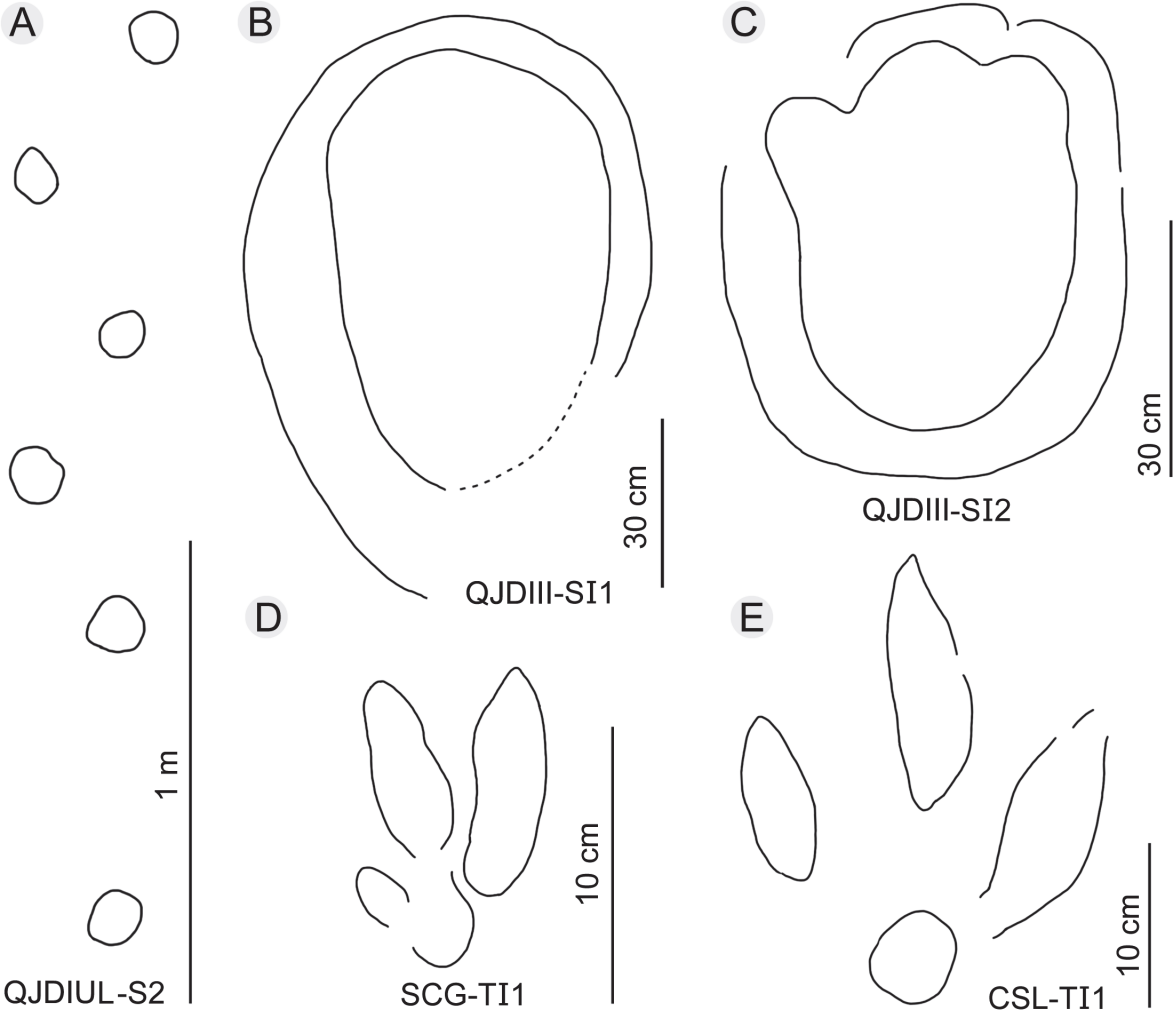
Interpretative outline drawing of sauropod and theropod trackway and tracks at Qianjiadian tracksites IU (A) and III (B–C), Shicaogou tracksite (D), and Changshouling tracksite (E).

At QJDILL (Fig 4) there are two partial trackway segments of ambiguous configuration (Figs 4 and 10): S1(two pairs of manus and pes prints), and S2 (two pes prints and three manus prints), plus at least 16 isolated tracks. QJDIUL (Fig 11) reveals three trackways S1–S3, preserved 2, 3, 57 tracks respectively, and 27 isolated tracks. QJDII site (Fig 12 has 15 isolated tracks. All tracks are preserved in situ. QJDIII (Fig 13B and 13C) has approximately 70 isolated tracks. All original specimens remain in the geopark.

According to the size, morphological characteristics and rotation angle of the manus, all tracks at the Qianjiadian tracksite can be divided into three morphotypes:

Morphotype A includes the QJDILL-S2 trackway (Fig 4, Table 2), the mean length of the pes is 53.5cm. Although the trackway is poorly-preserved, it possesses the morphological characteristics of a distinctly wide-gauge trackway. The manus impressions of QJDILL-S2 lie anteromedial to the pes impressions. The average TL/TW ratios of the manus and pes impressions are 0.7 and 1.5 respectively. Taking the best-preserved examples RP2 and RM2, the manus prints are U−shaped, whereas the claw marks and the metacarpophalangeal region are indistinct. The pes prints are oval, digits I–III claw marks are present, and the metatarsophalangeal region is smoothly curved. The manus impressions are rotated approximately 23° outward from the trackway axis, the pes impressions are rotated approximately 26° outward. The pace angulation of the manus traces is 138°.

**Table 2:** Measurements (in cm) of the sauropod trackways from the Qianjiadian tracksites, Yanqing, Beijing, China.

Number.	TL	TW	R	PL	SL	PA	L/W	WAP	WAP/P’TL	WAM	WAM/M’TW
QJDILL-S1RP1	34.0	27.0	32°	86.2	—	—	1.3	—	—	—	—
QJDILL-S1RM1	16.0	25.5	62°	104.5	—	—	0.6	—	—	—	—
QJDILL-S1LP1	40.5	27.0	24°	—	—	—	1.5	—	—	—	—
QJDILL-S1LM1	17.0	24.7	109°								
Mean-P	37.3	27.0	28°	86.2	—	—	1.4	—	—	—	—
Mean-M	16.5	25.1	86°	104.5	—	—	0.6	—	—	—	—
QJDILL-S2RP1	52.0	37.0	20°	—	271.5	—	1.4	—	—	—	—
QJDILL-S2RM1	24.5	30.4	23°	131.9	233.5	—	0.8	—	—	74.9	2.5
QJDILL-S2LP1	—	—	—	—	—	—	—	—	—	—	—
QJDILL-S2LM1	22.0	37.5	—	145.7	—	—	0.6	—	—	—	—
QJDILL-S2RP2	55.0	35.0	32°	—	—	—	1.6	—	—	—	—
QJDILL-S2RM2	23.5	30.5	—	—	—	—	0.8	—	—	—	—
Mean-P	53.5	36.0	26°	—	271.5	—	1.5	—	—	—	—
Mean-M	23.3	32.8	23°	138.8	233.5	—	0.7	—	—	74.9	2.5
QJDIUL-S1LP1	37.5	26.5	—	—	200.0	—	1.4	—	—	—	—
QJDIUL-S1LP2	37.5	27.0	—	—	—	—	1.4	—	—	—	—
Mean-P	37.5	27.0	—	—	200.0	—	1.4	—	—	—	—
QJDIUL-S2RP1	12.5	10.0	31°	—	64.0	—	1.3	—	—	—	—
QJDIUL-S2LP1	—	—	—	—	—	—	—	—	—	—	—
QJDIUL-S2RP2	12.0	11.0	46°	37.0	64.0	120°	1.1	18.9	1.6	—	—
QJDIUL-S2LP2	12.5	11.0	39°	36.0	65.0	119°	1.1	19.3	1.5	—	—
QJDIUL-S2RP3	11.0	9.0	39°	38.0	64.0	110°	1.2	23.1	2.1	—	—
QJDIUL-S2LP3	12.0	8.5	—	39.0	—	—	1.4	—	—	—	—
QJDIUL-S2RP4	11.5	11.0	—	—	—	—	1.0	—	—	—	—
Mean-P	11.9	10.1	39°	37.5	64.3	116°	1.2	20.4	1.7	—	—
QJDIUL-S3RP1	20.0	—	45°	50.0	85.5	118°	—	25.8	1.3	—	—
QJDIUL-S3RM1	—	—	—	48.0	90.5	—	—	—	—	—	—
QJDIUL-S3LP1	25.5	19.0	—	50.0	—	—	1.3	—	—	—	—
QJDIUL-S3LM1	11.0	14.0	—	55.0	—	—	0.8	—	—	—	—
QJDIUL-S3RP2	20.0	10.0	42°	—	95.0	—	2.0	—	—	—	—
QJDIUL-S3RM2	16.5	16.5	32°	—	92.0	—	1.0	—	—	—	—
QJDIUL-S3LP2	—	—	—	—	—	—	—	—	—	—	—
QJDIUL-S3LM2	—	—	—	—	—	—	—	—	—	—	—
QJDIUL-S3RP3	19.5	15.5	46°	53.0	94.0	115°	1.3	28.3	1.5	—	—
QJDIUL-S3RM3	14.5	12.5	—	48.0	—	—	1.2	—	—	—	—
QJDIUL-S3LP3	27.0	19.0	57°	54.0	95.0	125°	1.4	24.3	0.9	—	—
QJDIUL-S3LM3	10.5	16.5	53°	—	94.0	—	0.6	—	—	—	—
QJDIUL-S3RP4	22.0	14.5	27°	52.0	93.0	118°	1.5	27.2	1.2	—	—
QJDIUL-S3RM4	—	—	—	—	—	—	—	—	—	—	—
QJDIUL-S3LP4	19.5	19.5	67°	55.0	90.0	117°	1.0	26.9	1.4	—	—
QJDIUL-S3LM4	9.5	18.0	71°	58.0	94.0	128°	0.5	—	—	20.9	1.2
QJDIUL-S3RP5	23.5	17.0	24°	48.0	90.0	115°	1.4	27.1	1.2	—	—
QJDIUL-S3RM5	12.0	18.0	106°	46.0	86.0	130°	0.7	—	—	19.3	1.1
QJDIUL-S3LP5	22.0	17.5	56°	58.0	90.0	110°	1.3	30.0	1.4	—	—
QJDIUL-S3LM5	10.0	17.5	70°	49.0	87.0	130°	0.6	—	—	20.0	1.1
QJDIUL-S3RP6	21.5	15.5	28°	49.0	88.0	113°	1.4	29.0	1.3	—	—
QJDIUL-S3RM6	10.0	16.5	102°	46.0	87.0	129°	0.6	—	—	20.7	1.3
QJDIUL-S3LP6	23.0	21.0	41°	53.0	84.0	110°	1.1	28.7	1.2	—	—
QJDIUL-S3LM6	11.0	18.5	58°	50.0	82.0	121°	0.6	—	—	23.4	1.3
QJDIUL-S3RP7	25.5	15.0	34°	45.0	88.0	115°	1.7	26.4	1.0	—	—
QJDIUL-S3RM7	11.0	17.5	—	43.0	—	—	0.6	—	—	—	—
QJDIUL-S3LP7	22.5	17.5	57°	57.0	88.0	110°	1.3	29.2	1.3	—	—
QJDIUL-S3LM7	12.5	18.0	54°	—	84.0	—	0.7	—	—	—	—
QJDIUL-S3RP8	26.5	17.0	26°	46.0	88.0	114°	1.6	28.2	1.1	—	—
QJDIUL-S3RM8	—	—	—	—	—	—	—	—	—	—	—
QJDIUL-S3LP8	21.0	18.0	33°	54.0	86.0	110°	1.2	30.0	1.4	—	—
QJDIUL-S3LM8	11.5	20.0	45°	53.0	85.0	—	0.6	—	—	—	—
QJDIUL-S3RP9	23.5	14.5	46°	47.0	90.0	112°	1.6	29.0	1.2	—	—
QJDIUL-S3RM9	—	—	—	42.0	85.0	—	—	—	—	—	—
QJDIUL-S3LP9	24.0	18.0	42°	55.0	90.0	110°	1.3	30.4	1.3	—	—
QJDIUL-S3LM9	11.0	18.5	62°	52.0	88.0	127°	0.6	—	—	22.1	1.2
QJDIUL-S3RP10	23.0	16.5	27°	48.0	88.0	109°	1.4	30.6	1.3	—	—
QJDIUL-S3RM10	11.0	14.0	76°	46.0	90.0	124°	0.8	—	—	23.2	1.7
QJDIUL-S3LP10	21.5	18.0	45°	55.0	88.0	108°	1.2	31.2	1.5	—	—
QJDIUL-S3LM10	13.0	17.0	58°	55.0	89.0	124°	0.8	—	—	23.3	1.4
QJDIUL-S3RP11	25.0	16.5	34°	48.0	90.0	113°	1.5	28.8	1.2	—	—
QJDIUL-S3RM11	9.5	15.0	112°	45.0	87.0	129°	0.6	—	—	21.4	1.4
QJDIUL-S3LP11	25.5	20.5	38°	56.0	91.0	116°	1.2	28.3	1.1	—	—
QJDIUL-S3LM11	11.0	20.0	44°	53.0	91.0	126°	0.6	—	—	22.5	1.1
QJDIUL-S3RP12	27.0	18.0	45°	49.0	88.0	120°	1.5	25.0	0.9	—	—
QJDIUL-S3RM12	13.5	16.0	44°	49.0	91.0	127°	0.8	—	—	22.0	1.4
QJDIUL-S3LP12	20.5	18.5	26°	49.0	85.0	117°	1.1	25.0	1.2	—	—
QJDIUL-S3LM12	13.0	21.0	63°	52.0	83.0	117°	0.6	—	—	25.1	1.2
QJDIUL-S3RP13	19.0	13.5	36°	48.0	90.0	118°	1.4	26.4	1.4	—	—
QJDIUL-S3RM13	13.5	16.5	57°	43.0	87.0	122°	0.8	—	—	23.3	1.4
QJDIUL-S3LP13	17.5	18.5	27°	55.0	86.0	108°	0.9	30.7	1.8	—	—
QJDIUL-S3LM13	11.0	14.0	—	54.0	87.0	—	0.8	—	—	—	—
QJDIUL-S3RP14	25.0	21.0	35°	44.0	83.0	110°	1.2	28.9	1.2	—	—
QJDIUL-S3RM14	13.0	15.5	46°	44.0	84.0	—	0.8	—	—	—	—
QJDIUL-S3LP14	22.5	15.5	—	54.0	83.0	—	1.5	—	—	—	—
QJDIUL-S3LM14	9.5	15.5	—	50.0	—	—	0.6	—	—	—	—
QJDIUL-S3RP15	23.0	15.5	34°	45.0	83.0	—	1.5	—	—	—	—
QJDIUL-S3RM15	10.0	16.0	75°	—	82.0	—	0.6	—	—	—	—
QJDIUL-S3LP15	—	—	—	—	—	—	—	—	—	—	—
QJDIUL-S3LM15	—	—	—	—	—	—	—	—	—	—	—
QJDIUL-S3RP16	23.0	18.0	—	—	—	—	1.3	—	—	—	—
QJDIUL-S3RM16	10.0	15.0	—	—	—	—	0.7	—	—	—	—
Mean-P	22.7	17.1	39°	51.0	88.5	114°	1.4	28.1	1.3	—	—
Mean-M	11.6	16.7	65°	49.1	87.5	126°	0.7	—	—	22.1	1.3
SCG-SIP1	80.0	64.0	—	—	—	—	—	—	—	—	1.3
SCG-SIM1	50.0	—	—	—	—	—	—	—	—	—	—
CSL-S1LP1	51.0	32.0	—	100.0	—	—	—	—	—	—	1.6
CSL-S1RP1	50.5	34.0	—	—	—	—	—	—	—	—	1.5

Abbreviations: TL: Track length; TW: Track width; R: Rotation; PL: Pace length; SL: Stride length; PA: Pace angulation; L/W: length/ width; WAP: Width of the angulation pattern of the pes (calculated value); WAM: Width of the angulation pattern of the manus (calculated value); WAP/P'TL and WAM/M'TW are dimensionless.

Morphotype B contains the trackways QJDILL-S1, QJDIUL-S2, S3 (Figs 4, 10, 11, 13 and Table 2), of which QJDILL-S1 (Figs 4 and 10) is the best-preserved, with two pairs of tracks only. The mean pes length is 37.3 cm. QJDIUL-S3 is shallow but well-preserved with a mean pes length of 22.7 cm.

The centers of the pes tracks are positioned somewhat closer to the trackway midline than those of the manus tracks. In the trackways QJDILL-S1 and QJDIUL-S3, the manus tracks show a pronounced outward rotation relative to the midline (86° on average, ranging 62°–109°; 65° on average, ranging 32°–112°, respectively). On the contrary, the pes tracks are less outwardly rotated (28° on average, ranging 24°–32°; 39° on average, ranging 24°–67°, respectively).

In the QJDILL-S1 trackway, the manus tracks are oval, narrowing at mid-length, and the metacarpophalangeal pad region (i.e., the posterior border of the track) is concave. All of the better-preserved pes tracks have three broad, discernable digit impressions. For some tracks, such as LP1 and RP1, the middle digit trace of the pes appears longer than the outer digits, whereas digit I is usually broader and more robust than the other two digits. The metatarsophalangeal pad region is smoothly curved.

Morphotype C contains the pes-only trackway QJDIUL-S2 (Fig 13A) with a mean pes length of 11.9 cm, which is the smallest sauropod trackway at the Qianjiadian tracksites. The pes impressions are rotated approximately 39° outward from the trackway axis, similar to QJDILL-S2 and QJDIUL-S3. However, due to the lack of manus tracks, it is difficult to determine if QJDIUL-S2 should belong to Morphotype A or B. It is therefore tentatively assigned to Morphotype C. All pes prints are oval, without digital marks.

Two well-preserved but isolated tracks, QJDIII-SI1 and SI2 (Fig 13B and 13C), were found at tracksite III. SI1 is 70 cm in length, making it the largest track at the Qianjiadian tracksites. The track is well-preserved but lacks further details. The TL/TW ratio is 1.7, greater than that of QJDILL-S2. SI1 exceeds all previous medium-sized sauropod trackways in size, and probably belongs to Morphotype A. SI2 is 48.5 cm in length, the TL/TW ratio is 1.3. All three digit impressions are distinct, among which the most developed one is probably digit I. Although these tracks lack manual traces, the TL/TW ratio is consistent with Morphotype B, and are herein temporarily assigned to Morphotype B.

Tracksite III reveals four thin layers with sauropod tracks. The interval between the four thin layers ranges between 15–20 cm. QJDIII-SI1 at the upmost layer is the deepest track (approximately 7 cm). SI2 in the fourth layer is comparatively deep (approximately 3 cm). Other tracks are quite shallow, approximately 1 cm deep. Unlike ordinary undertracks, no corresponding track was discovered in the strata overlying the strata containing the tracks. This suggests that the tracks became shallower due to natural weathering, before being covered by sediments. On the other side, this could also indicate a difference in substrate consistency, such as the sediments dewatering over time and becoming more firm. Perhaps the trackmaker of SI1 left a deeper footprint on a surface of variable consistency, which was deep enough that, even after natural weathering, was still preserved with a moderate relief.

#### 2.2 Comparisons and discussion

*Brontopodus* [62] [63] is one of the most common and well-known Cretaceous *sauropod track types*. Previously, most Early Cretaceous sauropod tracks in East Asia have been attributed to wide gauge *Brontopodus* [64] or to narrow gauge *Parabrontopodus* [61]. Globally the former ichnogenus is more common in the Cretaceous [63].

Most sauropod trackways in China are wide- (or medium-) gauge and are therefore referred to the ichnogenus *Brontopodus* [64]. The Qianjiadian sauropod Morphotype A trackway is a typical wide gauge trackway. Morphotype A configurations are also consistent with the characteristics of *Brontopodus* type tracks from other regions [62], [63], [65]. These features are 1) wide-gauge; 2) pes tracks that are longer than wide, large, and outwardly directed; 3) U−shaped manus prints; and 4) a high degree of heteropody (relatively low ratio of manus to pes size).

The heteropody of the well-preserved Morphotype A tracks is 1: 2.2. This is close to *Brontopodus birdi* (1:3) but less than in the narrow-gauge ichnotaxa *Breviparopus* (1:3.6) or *Parabrontopodus* (1:4 or 1:5). The wide-gauge of the *Brontopodus*-type trackways suggests that the tracks were left by titanosauriform sauropods [66], [64], which is consistent with the brachiosaurid sauropod materials yielded from the middle section (the Second and Third Members) of the Tuchengzi Formation [7]. It suggests that large-size titanosauriform dinosaurs roamed in this region during the time interval across the Jurassic-Cretaceous boundary in Tuchengzi Formation [27].

Zhang et al. [20] assigned Morphotype B to thyreophoran tracks cf. *Deltapodus* isp. When Xing et al. [58] described medium-sized sauropod trackways from the Jishan tracksite, the differences between thyreophoran tracks and medium-sized sauropod tracks were further analysed. For example, the former usually lack a strong outward rotation of the manus, whereas sauropod tracks with a strongly outward rotated manus, as well as with three digital impressions of the pes, are common. Correspondingly, trackways of small sauropods from southern South Korea ([67]: Fig. 35.4A) have similar pace angulations: 110° for the manus, 125° for the pes.

Xing et al. [61, 68–70] assigned the narrow-gauge, medium-sized sauropod trackways from the Nanguzhai tracksite and the Jishan tracksite to *Parabrontopodus* isp. QJDILL-S1 is clearly narrow-gauge and medium-sized too, but the surface ratio of the manus:pes is ~1:2.2, which is larger than ~1:3.5 of the Jishan specimens. QJDIUL-S3 WAP/P’TL is 1.3, which indicates that it is between a medium-gauge and a wide-gauge trackway, and the surface ratio of manus:pes is ~1:2.0, which is also larger than in the Jishan specimens. Thus the Qianjiadian specimen is tentatively assigned to cf. *Parabrontopodus* isp.

This type of middle-sized sauropod tracks with strongly outward rotated manus tracks might have had a wider distribution in China. They are known for example from Malingshan, Jiangsu Province [61], Linshu, Shandong Province [70], Zhucheng [69], Zhongpu, Gansu [71] and Litan, Yanguoxia area, Gansu [72]. However, these tracksites are all from the Early Cretaceous, and the Qianjiadian Morphotype B is the stratigraphically oldest occurrence.

The associations of Qianjiadian Morphotype A and Morphotype B suggest that large-sized and medium-sized sauropods roamed the same area contemporaneously. They are similar to those of the Jishan and Yanguoxia tracksites, although the latter shows a much better preservation of tracks. Alternatively, this could indicate that two types of sauropod trackmakers occupied different ecological niches, both flourishing across the Jurassic–Cretaceous boundary interval and Early Cretaceous in northern, eastern, and northwestern China. Interestingly, no sauropod body fossils have been found in the area thus far.

Due to the lack of sufficient evidence, Morphotype C is presumed as a juvenile representative of Morphotype A or Morphotype B. Further discussion requires more material. Marty et al. [73] considered that pes-only trackways might frequently be regarded as trackways of relatively fast moving sauropods, where the pes overprints the manus (e.g. [74]). Another possibility is that the manus trace was too shallow to be preserved. According to Myers and Fiorillo [75], juvenile and adult sauropods may have adopted different feeding and herding strategies, resulting in their segregation, which is probably the cause of small-sized sauropod tracks being rare at the Qianjiadian tracksite.

### 3 Possible ornithischian tracks

At the QJDILL site there is one single poorly-preserved pace, QJDILL-O1L1 and OR1 (Figs 4, 8I and 8J). All original specimens remain in the geopark.

Ornithopod tracks are rare in the Tuchengzi Formation. Except for the Qianjiadian tracksite, only the Shangyi tracksite yielded a possible ornithopod specimen [50]. However, the possibility of it being a poorly-preserved theropod track cannot be excluded. The possible ornithopod tracks at the Qianjiadian tracksite are similarly poorly-preserved. The best-preserved track is QJDILL-O1L1 (only digit IV is damaged). The TL/TW ratio is 1.3. The TL/TW ratio of the anterior triangle is 0.33. The traces of digits II–IV are almost equal in the length. The trace of digit III has a strong and blunt claw or mark. The heel is triangular. There is no distinct border between the heel and the three digits. The divarication angle between digit II and digit IV is 61° (II 26°III 35°IV.). O1R1 lacks digit III, but the other digits are similar to O1L1 in morphology. The step of QJDILL-O1 is 4.3 × FL.

This track is slightly reminiscent of *Dinehichnus* yielded from the Upper Jurassic Morrison Formation of U.S.A. and the Lower Cretaceous of [54], [76], [77]. *Dinehichnus* is believed to have been left by a medium-sized ornithopod [54], which is similar to QJDILL-O1 in size (footprint length 10–19cm). However, the divarication is approximately 90°, which is larger than the 61° measured in QJDILL-O1, and the distal digit III is pointed in *Dinehichnus*, which is different from the round distal digit III of QJDILL-O1.

Previously ornithischian tracks had been reported from the Qianjiadian tracksite and attributed to thyreophora (cf. *Deltapodus*) [20]. These interpretations were preliminary and very likely incorrect. Thus, the further studies, outlined here, have failed to support this inference. Most of these possible thyreophoran tracks are here reinterpreted as sauropod tracks (see previous section). As noted below the possibility that certain other small tridactyl tracks are of ornithopod affinity is discussed and cannot be ruled out entirely. The possible documentation of either ornithopod or thyreophoran tracks, appears to be inconsistent with the general pattern of saurischian dominance in the Tuchengzi ichnofaunas.

### 4 Speed and body length of trackmakers

The SL/h of the Qianjiadian theropod trackways are 1.46–2.91 (Table 3), which suggests that the trackmaker speed varied in the area. The speed of the trackmakers is 4.36–13.43 km/h. Using the average h to body length ratio of 1:2.63 [14], the trackmaker of the Qianjiadian theropod Morphotype A tracks are small-sized (length 0.65m), medium-sized (length ~1.30–2.37 m) and large-sized (length 3.35–3.87 m); the trackmakers of Morphotype B tracks are medium-sized (length ~1.42–2.37 m) and large-sized (length 3.67 m).

**Table 3:** Estimated data of the speed of Yanqing theropod trackmakers.

No.	F = 4.5	
	SL/h	S (km/h)
QJDIUL-T1	2.16	8.57
QJDIUL-T2	1.46	4.36
QJDIUL-T3	2.69	12.24
QJDIUL-T4	2.70	12.31
QJDIUL-T8	2.46	9.04
QJDIUL-T10	2.01	7.96
QJDIUL-T12	2.91	13.43
QJDIUL-T13	2.22	9.07
QJDIUL-T14	1.67	5.22
QJDIUL-T15	1.74	6.19

Abbreviations: F, hip height conversion factors; SL/h, relative stride length; S, speed

For ornithopods, h is 4.8 times longer than the FL, using the ratio for small ornithopods proposed by Thulborn [41]. The hip height h of QJDILL-O1 is 0.66 m. Referring to the hip height to body length ratio of theropods, QJDILL-O1 body length is estimated as approximately 1.73 m.

For sauropods, Alexander [40] first suggested that h = 4×FL, whereas, later, Thulborn [41] estimated h = 5.9×FL. The SL/h of the Qianjiadian sauropod trackways are 0.66–0.92, 0.97–1.35 (Table 4) and accordingly suggests walking. Using the equation to estimate *v* from trackways [40], the mean *v* of the trackmaker is between 1.62–3.89 km/h and 2.56–6.12 km/h. The h of the Qianjiadian Morphotype A–C sauropod trackmakers would be approximately 3.2 m, 1.3–2.2 m, and 0.7 m, respectively. Body length: h ratio of *Shunosaurus* (a typical Middle Jurassic Chinese sauropod) is 3.7:1 (based on [39]: Fig 3). The body length of the Qianjiadian sauropod trackmakers are estimated to be approximately 11.7 m (Morphotype A), 5.0–8.2 m (Morphotype B), and 2.6 m (Morphotype C). For the isolated QJDIII-SI1, h is approximately 4.1 m and the body length is approximately 15.3 m.

**Table 4:** Estimated data of the speed of Yanqing sauropod trackmakers.

No.	F = 5.9		F = 4	
	SL/h	S (km/h)	SL/h	S (km/h)
QJDILL-S2	0.86	3.89	1.27	6.12
QJDIUL-S1	0.90	3.53	1.33	5.58
QJDIUL-S2	0.92	2.05	1.35	3.20
QJDIUL-S3	0.66	1.62	0.97	2.56

Abbreviations: F, hip height conversion factors; SL/h, relative stride length; S, speed.

## Shicaogou Tracksite, Yanqing County

The Shicaogou Tracksite is located at the southwest of Tracksite I in the Third Member of the Tuchengzi Formation. Characteristic features of the surfaces are ripple marks and invertebrate traces (cf. *Monocraterion* isp.). Six sauropod tracks and one theropod track have been observed. One pair of sauropod manus and pes prints are well-preserved and were catalogued as SCG-SIP1 and SIM1. The tracks were preserved on the rippled surface. SIP1 is an oval pes trace, approximately 80 cm in length, and with a TL/TW ratio of 1.3. There is a boundary between digit I–IV impressions. The manus is incomplete and roughly round. The diameter is approximately 50 cm, similar to *Brontopodus* type tracks in morphology. Based on the formula described above, the hip height of SCG-SIP1 is estimated at approximately 4.7 m, the body length at approximately 17.5 m.

One single possible didactyl track is catalogued as SCG-TI1 (Fig 13D). The length is 10.8 cm and the TL/TW ratio is 2. Digit II is a short, round impression. Compared with digits III and IV, the impression of digit II is more shallow and indistinct. It is located proximo-medially to digit III. Digit IV is longer than digit III. The metatarsophalangeal pad is located near the proximal axis of digit III and is sub-round in shape. The divarication angle is 27° between digits III–IV. The heading orientation and further imprints were covered by sediments, although future excavation will hopefully reveal further tracks of the trackway.

The two digit impressions could be interpreted as traces of digits III and IV of a deinonychosaurian track [78]. According to the size reported by Xing et al. [14], the mean pes length of the small-sized tracks is 10 cm. CSL-TI1 is within this size range. CSL-TI1 resembles *Dromaeosauripus yongjingensis* [79] in morphology, but the divarication is larger than that of *D*. *yongjingensis* [79]. Given that it might rather be assigned to *Menglongipus sinensis *[14], the presence of this ichnotaxon in the Tuchengzi Formation, CSL-TI1 would be a new record, or at least tentative support for a wider distribution of this ichnotaxon. However, Gaston et al. [80] suggest that some poorly-preserved tridactyl tracks may lack the distal parts of digit II and become a “didactyl” variant. By one isolated track it is difficult to determine whether it represents an actual didactyl track or an extramorphological variant created by a particular behavior of the track maker, by the influence of the substrate, or by a combination of both.

## Changshouling Tracksite, Yanqing County

The Changshouling tracksite is located northeast of Tracksite 1. Stratigraphically it is positioned at the top of the third Member of the Tuchengzi Formation. The tracks are preserved on purple argillaceous siltstone. Three sauropod and theropod tracks were observed.

One isolated theropod track is catalogued as CSL-TI1 (Fig 13E), which is 28 cm in length and the TL/TW ratio is 1.4. Digit II is the shortest, digit III nearly equals digit IV, and digit III has a sharp claw mark. TI1 resembles Qianjiadian Morphotype A in morphology, but the developed metatarsophalangeal pad, its morphology and position, make it more similar to *Asianopodus*.

Two sauropod tracks CSL-S1LP1 and S1RP1 constitute one single pace. The mean length of the tracks is 51 cm. Both tracks left displacement rims of approximately 10 cm in width. The tracks are seriously weathered. No morphological details support their assignment to a specific ichnogenus.

## Paleoecology

Overlapping tracks were observed in sauropod and theropod trackways of Qianjiadian tracksite 1U. Sauropod trackway S3 has disturbed partial tracks of the theropod trackway T12 (Fig 11). For example, the posterior portion T12R3 is overprinted, and T12R2 digit III has been deformed. The latter suggests a short time interval between the formation of S3 and T12, when the latter track was not entirely solidified.

Especially in Asia, sauropod tracks generally occur in semiarid-arid environments of fluvial facies or lacustrine facies of inland sedimentary basins [64]. The tracksites with rich footprints in North China, (e.g. the Yanguoxia tracksite) are dominated by sauropod, theropod, ornithopod, pterosaur and bird tracks [81], whereas the Chabu tracksite contains sauropod, theropod and bird tracks [82], the Linshu tracksite contains sauropod, theropod, and probable psittacosaur tracks [70]. In general, in the area where sauropods flourished, ornithopod tracks are rare. This pattern is seen in the Qianjiadian tracksite, which preserves abundant theropod and sauropod tracks, but relatively few ornithopod tracks.

In the Yanqing area representing the Jurassic-Cretaceous boundary sequence, abundant theropod (including dromaeosaur), bird, sauropod, and possible ornithopod tracks have been found. The bone record includes basal ceratopsians as well [5], [6]. Both ichno- and bodyfossils suggest the presence of a diverse dinosaur fauna during deposition of the Tuchengzi Formation.

## Conclusions

Theropod and sauropod trackways from the Jurassic-Cretaceous boundary Tuchengzi Formation support the former view of saurischian dominated assemblages in East Asia during this time interval [56]. This might partly be a preservational and taphonomic effect. Most abundant are theropod tracks similar to those of the *Grallator–Eubrontes* plexus and well known from Lower Jurassic strata. The presence of ornithopods is possibly indicated by isolated occurrences but remains a rather scarce element. Theropod trackmakers were small to medium-sized individuals that probably belonged to different taxonomic groups of coelurosaurs including functionally didactyl dromaeosaurs. The sauropod trackways belong to medium and large-sized animals that represent both narrow-gauge and wide forms, the latter being attributed to titanosauriforms whose skeletons are known from the same unit. The track-record from the Tuchengzi Formation is important because it supplements the scarce bone record that thus far lacked theropods. Stratigraphically it fills a gap in the dinosaur record representing strata that are slightly older than the famous Jehol Biota from Liaoning Province.
